# “Non-Essential” Proteins of HSV-1 with Essential Roles In Vivo: A Comprehensive Review

**DOI:** 10.3390/v13010017

**Published:** 2020-12-23

**Authors:** Christos Dogrammatzis, Hope Waisner, Maria Kalamvoki

**Affiliations:** Department of Microbiology, Molecular Genetics, and Immunology, University of Kansas Medical Center, Kansas City, KS 66160, USA; cdogrammatzis@kumc.edu (C.D.); h721w416@kumc.edu (H.W.)

**Keywords:** HSV-1 non-essential proteins, HSV-1 egress, HSV-1 envelopment, innate immunity, HSV-1 based therapies, gene silencing

## Abstract

Viruses encode for structural proteins that participate in virion formation and include capsid and envelope proteins. In addition, viruses encode for an array of non-structural accessory proteins important for replication, spread, and immune evasion in the host and are often linked to virus pathogenesis. Most virus accessory proteins are non-essential for growth in cell culture because of the simplicity of the infection barriers or because they have roles only during a state of the infection that does not exist in cell cultures (i.e., tissue-specific functions), or finally because host factors in cell culture can complement their absence. For these reasons, the study of most nonessential viral factors is more complex and requires development of suitable cell culture systems and in vivo models. Approximately half of the proteins encoded by the herpes simplex virus 1 (HSV-1) genome have been classified as non-essential. These proteins have essential roles in vivo in counteracting antiviral responses, facilitating the spread of the virus from the sites of initial infection to the peripheral nervous system, where it establishes lifelong reservoirs, virus pathogenesis, and other regulatory roles during infection. Understanding the functions of the non-essential proteins of herpesviruses is important to understand mechanisms of viral pathogenesis but also to harness properties of these viruses for therapeutic purposes. Here, we have provided a comprehensive summary of the functions of HSV-1 non-essential proteins.

## 1. Introduction

The large family of DNA viruses, *Herpesviridae*, have co-evolved with mammals for millions of years [1,2]. The family *Herpesviridae* is further divided into three subfamilies, *Alphaherpesvirinae*, *Betaherpesvirinae*, and *Gammaherpesvirinae.* Herpes simplex virus type 1 (HSV-1), a member of *Alphaherpesvirinae*, is one of the most well-studied representatives of this family of viruses and will be the focus of this review. 

HSV-1 is an enveloped dsDNA virus, which has a genome size of about 152 kb and virion size of about 150–300 nm in diameter [3]. The virion contains the envelope decorated with viral glycoproteins and a proteinaceous layer known as the tegument, which surrounds the capsid of the virus containing the genome. HSV-1 is an important human pathogen, with approximately 80% of the human population infected [3]. Symptoms of HSV-1 infection vary, from lesions in the oral-facial region (“cold sores”), to herpes keratitis, the leading cause of infectious blindness, to herpes encephalitis, which can be fatal. When HSV-1 encounters a host, it will first infect the mucosal epithelial cells in the oral-facial region (although HSV-1 also causes genital infections). It is in these cells that the virus undergoes lytic replication. The virus will then infect innervating sensory neurons, travel anterograde to the trigeminal ganglia (TG), and establish latency, where it will remain for the life of the infected individual. When HSV-1 undergoes latency there are very few genes expressed, including an 8.3-kb region known as the latency-associated transcript (LAT), which is a long non-coding regulatory RNA spliced to about 1.5- and 2-kb introns that have regulatory roles on viral genes expression, and a 6.3-kb exon encoding multiple microRNAs, which target many of the IE genes and other lytic genes, thus suppressing viral replication [4,5,6,7,8,9,10]. It seems that LAT may be important for reactivation of HSV-1 from latency and for blocking apoptosis [11,12,13,14,15,16,17,18]. Periodically, HSV-1 will reactivate from latency due to stress, immunosuppression, or other stimuli, and newly produced virions will travel retrograde to the initial site of infection. There is currently no cure for HSV-1 and no vaccine. 

There are three classes of viral genes for HSV-1 and they are expressed in a cascade fashion [19,20]. The virus first encodes the immediate-early (IE) or alpha (α) genes (*ICP0, ICP4, ICP27, ICP22,* or *ICP47*) whose products are important for expression of the next class of viral genes, the early (E) or beta (β) genes. The early genes encode proteins largely involved in viral DNA replication and, along with the immediate-early genes, facilitate the expression of the late class of viral genes. The late (L) or gamma (γ) class of viral genes express proteins involved in virion assembly and egress. HSV-1 genes are also divided based on stretches of unique sequences in the genome. Therefore, there are a class of unique long (U_L_) and unique short (U_S_) genes depending on which region of the genome the gene is expressed from. These unique regions are flanked by inverted repeats. Thus, the HSV-1 genome is structured as follows: TR_L_-U_L_-IR_L_-IR_S_-U_S_-TR_S_. There are about 58 U_L_ genes and about 13 U_S_ genes that have been characterized for functionality, though there are more viral genes that have not been well characterized or described (Dolan 1998). 

Interestingly, although HSV-1 is known to encode 80 genes, it has also been found that about half of these genes are non-essential for viral replication in cell culture [21,22]. Essential genes of HSV-1 are involved in viral DNA replication, the transcription of certain viral genes, genes encoding capsid proteins, genes encoding viral DNA packaging proteins, and some envelope glycoproteins. HSV-1 genes determined to be non-essential are involved in nucleic acid metabolism, combating various host responses to infection, facilitating optimal viral replication, facilitating primary envelopment, virus pathogenesis, or other functions that are not yet characterized (Table 1). While deletion of the non-essential genes in cell culture does not inhibit viral replication, these genes are generally essential for replication in the natural human host as mutant viruses deleted of non-essential genes have rarely been isolated from a patient. One example are mutants in the viral glycoprotein gC that have been recovered from patients with recurrent herpes keratitis [23,24].

It is of great interest to understand the roles of non-essential genes to better understand virus–host interactions. Moreover, the non-essential genes have properties that make them attractive for the development of therapeutics. There are varying degrees of deficiency of viruses mutated for non-essential genes when grown in cell culture, and for some of these genes, the defect is cell type specific [25,26]. There is still much to learn about the non-essential genes of HSV-1. Here, we present a comprehensive analysis of the current understanding of the roles of non-essential genes of HSV-1. We explore the functions ascribed to these genes and their corresponding proteins, the potential treatment and therapeutic avenues that can be explored based on the functions and characterization of select HSV-1 non-essential genes, and the complex and intricate roles of non-essential genes in HSV-1 infection.

## 2. Repressors of Gene Silencing, Viral Transactivators, and Host Evasion Factors

### 2.1. RL2 or α0 (ICP0)

The infected cell protein 0 (ICP0) of HSV-1 was first reported as a nuclear phosphoprotein with an essential role in cell cultures only at low multiplicity of infection (MOI). ICP0 was deemed to be non-essential at high multiplicities of infection in cell cultures, but viral gene expression was reduced [19,27,28,29,30,31,32,33]. In certain cell lines, particularly cancer cell lines, such as the human osteosarcoma (U2OS), an ICP0-null virus replicates as efficiently as wild-type virus, which may be due to impaired recruitment of antiviral factors to the sites of viral gene transcription and DNA replication and/or due to lack of certain restriction factors [26,28,30,32,34,35]. Genes coding for ICP0 are present in the genomes of simplex and varicelloviruses, but they are absent from the mardivirus genus. These proteins show strong sequence homology to ICP0 within the RING (Really Interesting New Gene) finger domain. Orthologs of ICP0 are also present in lymphocryptoviruses (e.g., EBV) and the cytomegalovirus (CMV) [36,37,38]. The functions of ICP0 are broad, from activation of transcription and chromatin remodeling, to evasion of antiviral responses, cell cycle effects, interfering with DNA damage responses, and endocytosis. 

In early studies, ICP0 was found with ICP4 to stimulate ICP8 expression in transfection assays [39,40]. Furthermore, it was shown to function as a potent transactivator of different genes introduced into cells by transfection or infection, including the viral thymidine kinase (TK) gene and ICP6 gene, the human immunodeficiency virus (HIV) LTR, and several human papillomavirus (HPV) genes [41,42,43,44,45,46,47,48,49,50]. In fact, ICP0 was found to stimulate the expression of all three classes of HSV genes [31,51]. Therefore, ICP0 was proposed to be a promiscuous transactivator of gene expression.

**Figure 1:**
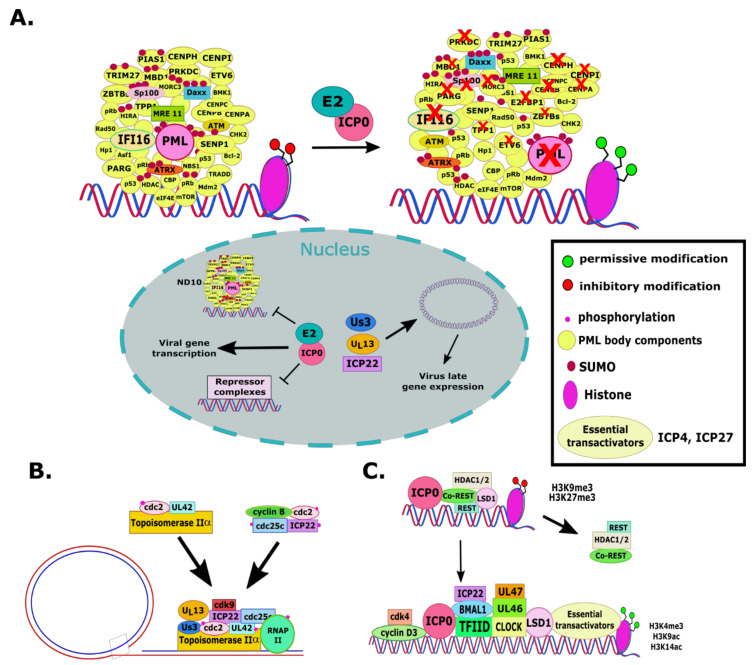
Nuclear functions of non-essential HSV-1 proteins. HSV-1 encodes multiple proteins able to counteract antiviral host responses within the nucleus. (**A**): ICP0 functions as an E3 ligase ubiquitin ligase that degrades ND10 components that encapsulate the viral genome in the nucleus, including PML, Daxx, SP100, centromeric proteins, and others. The degradation of IFI16 involves multiple factors. These events facilitate initiation of viral gene transcription. (**B**): The viral protein ICP0 is also known to disrupt repressor complexes that silence the viral genome, as well as recruit factors to enable viral gene transcription. Altogether, ICP0 facilitates permissive histone modifications, while suppressing silencing modifications, to enable for viral gene expression. (**C**): The viral kinases U_S_3 and U_L_13, with ICP22, are known to facilitate viral late gene expression, which occurs through the recruitment of host factors, such as Topoisomerase IIα and RNA polymerase II, to the sites of DNA replication in the nucleus. Together, these non-essential viral proteins are important for optimal expression of other viral genes and for viral DNA replication.

ICP0 also functions as an E3 ubiquitin ligase and most substrates ubiquitinated by ICP0 appear to be targeted for degradation (Figure 1A). This activity of ICP0 was mapped to residues 116–156, where there is a Zn^2+^-binding RING finger domain [52,53,54,55]. To exert its E3 ubiquitin ligase function, ICP0 forms a complex with different ubiquitin conjugation enzymes, including UbcH5a and UbcH6 [52,56,57,58,59,60]. Major targets of ICP0 are components of the nuclear domain 10 (ND10) bodies. As a DNA virus, the genome of HSV-1 transcribes and replicates in the nucleus. The host attempts to block viral gene expression and replication by entrapping the viral DNA in promyelocytic leukemia (PML)-nuclear bodies (NBs) and depositing histones and other repressor complexes on it. The main protein that orchestrates the formation of ND10 bodies is the PML. Other components of ND10s include the Sp100, Daxx, Mre 11, ATM, ATRX, p53, and others. ICP0 disrupts the ND10s by causing degradation of the different isoforms of PML, Sp100, and potentially of other proteins (Figure 1A) [34,59,61,62,63,64,65,66,67,68,69,70,71,72,73,74,75,76,77] Notably, several components of the ND10 bodies are interferon inducible genes, which underscores the synergy between gene silencing mechanisms and innate immunity in suppressing HSV-1 gene expression. ICP0-null viruses or E3 ubiquitin ligase mutants have viral DNA entrapped in PML-NBs at low MOI and display reduced transactivation activity and ability to block antiviral responses [34,64,78,79,80,81,82,83]. ICP0 E3 ligase-deficient viruses are hypersensitive to interferon, replicate poorly, and fail to reactivate efficiently from neuronal latency [25,55,67,68,69,70,71]. Based, on these observations, Dr. Kalamvoki’s group recently developed a high-throughput assay to screen for ICP0-E3 ubiquitin ligases inhibitors [72]. This assay is proximity based and takes advantage of the fact that ICP0 is autoubiquitinated and degraded during infection and that this ICP0 autoubiquitination can occur in vitro using the purified protein encoded by the exon II of ICP0 (contains the RF domain), UbcH5a, and Ub [60,73,74]. Screening a small compound library, Dr. Kalamvoki’s group identified potential scaffolds that can interfere with the ICP0 E3 ubiquitin ligase activity [72]. 

ICP0 has seven SUMO-interacting motif (SIM)-like sequences (SLSs), and multiple ND10 components, including PML and SP100, are SUMOylated; therefore, ICP0 could bind to them (Figure 1A) [34,56,75,76,77,78,79,80,81]. It has been found that inhibition of cellular ubiquitination led to an increase of SUMOylated proteins that ended up accumulating at PML-NBs [82]. ICP0 utilizes both SUMO-dependent and SUMO-independent mechanisms to degrade Sp100 and multiple PML isoforms in an effort to prevent restriction of the virus by the host [34,56,75,76,78,79,83,84]. Other proteins could also be the target of SUMO-dependent degradation by ICP0 [75,77,81]. Specifically, SUMO-dependent degradation of MORC3 by ICP0, which associates with Sp100, has been observed and this occurs in a RING-finger-dependent manner and appears to diminish the association of PML-NBs with viral DNA [85]. Additionally, there has been a function ascribed to ICP0 SUMO–SIM interactions at the ND10s to modulate the DNA damage response (DDR) during infection [86,87]. For example, the DNA repair function of the DNA-dependent protein kinase (DNA-PK) is inhibited by ICP0 through degradation of its catalytic subunit and this facilitates virus replication [88,89,90]. Additionally, ICP0 mediates the degradation of two E3 ubiquitin ligases RFN8 and RFN168 that act as mediators of the ATM pathway and trigger recruitment of downstream effectors to sites of double-strand DNA breaks [91,92,93,94,95]. More work will need to be done to characterize the degradation of SUMOylated proteins by ICP0 that are not related to the ND10s. The ability for ICP0 to interrupt SUMO interactions and to degrade SUMOylated proteins during infection is likely a strategy to modify the cellular proteome to both prevent antiviral responses and promote the infection [34,76,83].

In tandem with the dispersion of ND10 bodies, ICP0 activates the viral chromatin (Figure 1B). Immediately after its release in the nucleus, HSV-1 DNA associates with repressive histones and other repressor complexes [96,97,98]. However, markers of active gene expression label the viral chromatin during lytic infection, such as tri-methylation of histone H3 at lysine 4 (H3K4) and acetylation of H3 at lysine 9 and lysine 14 [97,98,99], while suppressive epigenetic modifications of histone H3 (H3K9me3 and H3K27me3) are removed in an ICP0-dependent manner (Figure 1B) [65,100,101]. ICP0 was also found to associate with class II HDACs in vitro and control their repressor activity [102,103]. In addition, ICP0 seems to promote histone acetylation, as demonstrated using inhibitors of histone deacetylases [103,104,105]. This is also supported by the fact that ICP0 recruits to the viral genome the histone acetyltransferase CLOCK through interaction with the circadian regulator protein BMAL1. This leads to recruitment of additional viral transactivators ICP4, ICP22, ICP27, and part of the host transcription complex TFIID [106,107,108]. Tandemly, ICP0 disrupts repressor complexes, such as the REST/CoREST/HDAC complex and LSD1 [109,110,111,112]. ICP0 disperses the REST/CoREST/HDAC1/2/LSD1 through interaction with CoREST in an effort to promote HSV-1 gene expression and DNA replication (Figure 1B) [78,112,113,114,115]. It was also found that the interferon-inducible gene 16 (IFI16), Daxx, and ATRX proteins serve to restrict the virus, likely through sensing of viral DNA and obstructing replication and causing deposition of silencing histone H3 [34,65,100,116,117,118,119,120]. ICP0 induces the degradation of ATRX and IFI16 [121,122,123,124,125]. Degradation of ATRX seems to be secondary to PML degradation, while depletion of ICP0 appears to be both ICP0 dependent and independent.

ICP0 has also been shown to harness cell cycle components to support the infection. Thus, ICP0 was found to recruit cyclin D3 and the kinase cdk4 to ND10s to enable viral gene transcription and DNA replication, which was also supported by the fact that ICP0 nuclear-to-cytoplasmic translocation was enabled by cyclin D3 (Figure 1B) [108,126,127]. ICP0 has been found to arrest cells in the G2/M phase to promote virus replication by activating the checkpoint kinase 2 (Chk2) [128]. Consistent with these roles of ICP0, it was also found to degrade the centromere proteins CENP-A, CENP-B, and CENP-C, inducing the interphase centromere damage response (iCDR) (Figure 1A) [129,130,131,132]. In addition to this disturbance to the cell cycle, it has been found that ICP0 degrades the DNA-interacting protein TPP1, leading to transcription of telomere repeat-containing RNA activation (TERRA) and increased viral replication [133].

**Figure 2:**
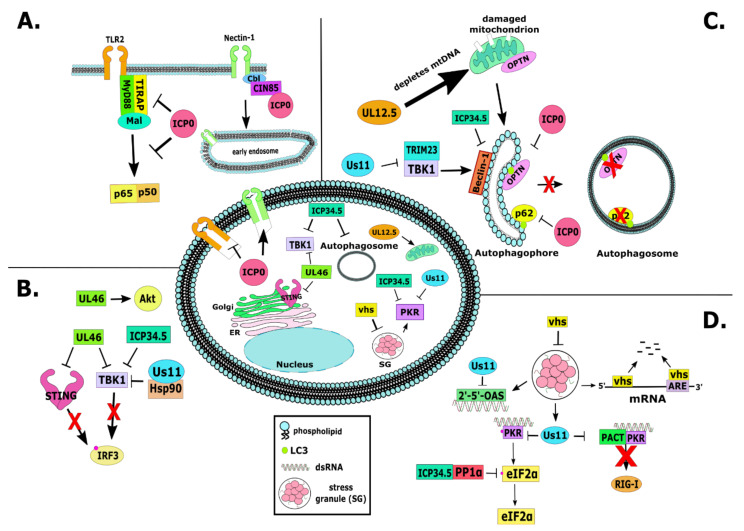
Cytoplasmic functions of some non-essential HSV-1 proteins. (**A**): ICP0 participates in two major functions in the cytoplasm. First, ICP0 degrades the TLR2 adaptors TIRAP and Mal, thus blocking NF-κB activity. ICP0 also binds to the endocytosis adaptor CIN85 and along with Cbl promotes internalization of the viral entry receptor Nectin-1. This is a mechanism to promote progeny virus spread to uninfected cells. (**B**): The tegument protein U_L_46 blocks STING and TBK1, which prevents stimulation of interferon-regulated genes. ICP34.5 and U_S_11 are also involved in blocking TBK1, emphasizing the importance of blocking TBK1 activity during HSV-1 infection. (**C**): The autophagy pathway is blocked during HSV-1 infection through binding of ICP34.5 to Beclin-1, thus preventing maturation of the autophagophore to an autophagosome. ICP0 has also been found to cause downregulation of p62 and OPTN proteins during infection, which may also serve as another mechanism of blocking selective autophagy. It has also been found that the protein encoded by U_L_12.5 causes depletion of mtDNA during infection, which causes damage to mitochondria. (**D**): HSV-1 prevents host translational shutoff from occurring during infection. One mechanism is through ICP34.5 binding to both PP1a and eIF2α, causing dephosphorylation of eIF2α and preventing shutoff of translation. HSV-1 also encodes vhs, which is a viral RNase that degrades AU-rich element (ARE) containing mRNAs. It has also been shown that vhs prevents the formation of cytoplasmic stress granules (SGs) during infection, which contain dsRNA that would otherwise cause PKR activation. HSV-1 also encodes U_S_11, which blocks PKR, thus blocking host translational shutoff and innate immunity activation, as well as blocking PKR and PACT-induced activation of RIG-I during infection.

As mentioned earlier, there are interwoven relationships between gene silencing and innate immunity and it is not coincidence the ICP0 targets them both. ICP0-null and other ICP0 mutant viruses displayed increased sensitivity to interferon both in vivo and in vitro [32,70,134,135]. As discussed above, ICP0 blocks the nuclear pattern recognition receptors (PRRs) IFI16 and DNA-PKs, which may also impact the cGAS and STING DNA sensing pathway [119,122,123,124,136,137]. Inhibition of STING-dependent immune responses involves ICP0 as ICP0-null virus growth is partially rescued in cells with impaired STING signaling [25,26]. Furthermore, ICP0 was found to reduce the levels of the Toll-like receptor 2 (TLR2) adaptors MyD88 (myeloid differentiation factor 88) and the Mal (MyD88 adaptor-like protein) TIRAP (TIR domain-containing adaptor protein), thus blocking immune responses through this pathway (Figure 2A) [138]. Overall, ICP0 has been proposed to inhibit IRF3 and IRF7-dependent immune responses to sequester these proteins away from host chromatin [139,140,141]. ICP0 was also recently found to have a role in autophagy inhibition through causing the downregulation of p62/SQSTM1 and OPTN autophagy adaptor proteins in a proteasome-dependent and RING finger-independent mechanism (Figure 2C) [142]. It was also demonstrated that the cytoplasmic ICP0 is most likely involved in this function. Another target of ICP0 is the deubiquitylating enzyme USP7 (ubiquitin-specific protease 7) or HAUSP. USP7 appears to bind and stabilize ICP0, but ICP0 degrades USP7 late during infection in a RING finger-dependent manner [58,78,79,143,144]. One reason why the virus could promote degradation of USP7 is because it has a major role in TLR- and TNFa receptor (TNFR)-induced gene expression [145].

Most functions of ICP0 discussed above are performed while in the nucleus. However, ICP0 translocates to the cytoplasm after enabling viral gene expression, where it remains for the reminder of the infection. The cytoplasmic functions of ICP0 remain unexplored. Dr. Roizman’s group first described an interaction of ICP0 with the endocytosis adaptor CIN85 [146]. Dr. Kalamvoki’s group has built upon these findings and reported that ICP0 promotes endocytosis of the viral entry receptor Nectin-1 (Figure 2A) [147]. This is perhaps a mechanism that ensures spread of progeny viruses to uninfected cells. CIN85 forms a complex with the Cbl E3 ligase that is involved in endocytosis of multiple surface components. Thus, ICP0 through CIN85 and Cbl could modulate the surface of infected cells to suppress antiviral responses.

Finally, the role of ICP0 has also been investigated during the latent stage of the virus. ICP0 appears to be important for efficient virus reactivation from latently infected trigeminal ganglia (TG) in mouse ocular infections [67,68,71,148,149,150,151]. ICP0 is also required for VP16-dependent viral reactivation [67,152]. While ICP0 is important for balancing lytic and latent infection, it is still not fully understood what its specific role is in this process.

### 2.2. U_L_46 and U_L_47 (VP11/12 and VP13/14)

The HSV-1 genes U_L_46 and U_L_47 were first described to encode proteins that modulate U_L_48 function [153,154,155]. It was then determined that UL46 encodes the phosphoprotein, VP11/12, and U_L_47 encodes the phosphoprotein VP13/14, which are also glycosylated, and both are non-essential in cell culture, although they have roles in enhancing the transactivation of viral genes by VP16 [156,157,158,159,160,161]. It was also found that U_L_47, but not U_L_46, deletion mutants of HSV-1 demonstrated a significant defect in alpha-TIF (U_L_48)-mediated expression of the TK gene, suggesting a supportive role in transcription of viral genes [161]. Homologs of U_L_46 and U_L_47 have been identified in multiple alphaherpesviruses, including pseudorabies virus, HSV-2, Marek’s disease virus, and VZV [153,157,162,163,164]. Homologs of U_L_47 have been found in bovine herpesvirus and equine herpesvirus, and U_L_47 has been found to be abundantly expressed in the viral tegument [153,165,166]. T cell responses specific to U_L_46 and U_L_47 have both been found in patients with intraocular HSV-1 infection [167].

VP11/12 has since been found to be tyrosine phosphorylated during HSV-1 infection in multiple lymphocyte cell types but not in epithelia or fibroblasts, and this was found to at least partly be due to the activity of lymphocyte-specific Src family kinase, Lck [168]. It was then found that HSV-1 infection increases the amount of phosphorylation of Lck at Y394 in Jurkat T cells, which occurred in a U_L_46-dependent manner [169]. Moreover, it was found that the Akt pathway is activated through the PI3 kinase, and U_L_46 was found to be involved in the activation of this pathway in HSV-1-infected HEL fibroblast cells (Figure 2B) [170,171]. VP11/12 was then found to bind to the Src family kinases Grb2, p85, and Shc, thus leading to Akt activation in a T cell lymphoma cell line, and in support of this, it was found that infection with U_L_46-deficient viruses in HFFs led to reduced Akt activation as compared to infection with wild-type virus [163,172]. In neuronal cells, it was also found that VP11/12 was necessary but not sufficient during infection to induce the phosphorylation and activation of the small GTPase Dynamin 2 [173]. U_L_46 has also been found to interact with the small GTPase Rab27a during HSV-1 infection of oligodendrocytes, as well as with gH and gD, but the significance of these interactions has not yet been investigated farther than reduced viral growth and infectivity in Rab27a-depleted cells [174].

It was demonstrated that HSV-1 infection of T cells led to the phosphorylation and degradation of adaptor complex protein Dok-2, which is involved in T cell regulation [175,176]. The degradation of Dok-2 occurred in a U_L_46-dependent manner, but the significance of this degradation has not yet been fully elucidated beyond a potential immune evasion strategy [177]. It was also recently found that U_L_46 works with Us3 to activate mTORC1 in fibroblasts, which supports virus growth [178]. Further supporting a role for U_L_46 in both immune evasion and viral growth was the observation that U_L_46 interacts with the DNA sensor STING (STimulator of INterferon Genes) at its C-terminus, which prevents the induction of interferon stimulatory genes and blocks type I interferon response to infection in fibroblasts (Figure 2B) [179]. Furthermore, it was found that U_L_46 binds to TBK1, TANK-binding kinase 1, at its N-terminus and this was found to also play a role in blocking the interferon pathway in fibroblasts (Figure 2B) [179,180]. In support of this, the pseudorabies homolog of U_L_46 was found to interact with STING [181]. Together, these results show strong evidence for a role of U_L_46 in blocking innate immune responses to HSV-1 infection.

Less work has been done to characterize U_L_47 or its gene product, VP13/14. It has been found that VP13/14 is involved in nuclear egress through interactions with the viral proteins UL34, UL31, and U_S_3 [182]. U_L_47 was observed in the nucleus of infected cells during early times post-infection, which is consistent with the interaction of U_L_47 with U_L_48, but was also detected in the cytoplasm at late times post-infection [183,184,185].

U_L_47 has also been found, with ICP27, to regulate mRNA processing and transport by redistributing polyadenylate binding protein (PABP) to the nucleus during infection of HeLa cells, further supporting a role for U_L_47 in modulating post-transcriptional processing of mRNAs [186]. U_L_47 has also been implicated in the regulation of the viral RNase vhs, which is discussed later in this review [187]. More work is needed to characterize fully the role of VP13/14 during HSV-1 infection, but so far it seems that U_L_47 facilitates viral infection by supporting viral gene transcription, mRNA processing, and transport.

### 2.3. U_L_49 (VP22)

**Figure 3:**
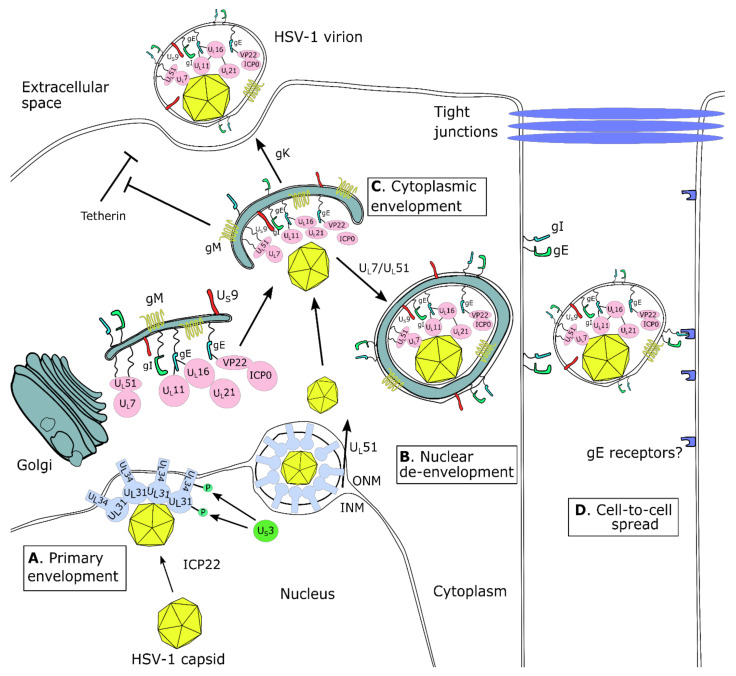
HSV-1 egress and relevant non-essential proteins. Essential proteins are not depicted. (**A**) U_L_31 and U_L_34 form the Nuclear Egress Complex (NEC), which drives the vesiculation of the inner nuclear membrane (INM) and primary envelopment of HSV-1 capsids. (**B**) Nuclear de-envelopment is mediated by U_L_51. Tegument and envelope proteins assemble in a complex network on membranes derived from the trans-Golgi network. (**C**) HSV-1 capsids undergo cytoplasmic envelopment in a process regulated by multiple non-essential proteins, which involves functional redundancy. After cytoplasmic envelopment, enveloped virions are sorted to the extracellular space, which requires gK. HSV-1 release from the cell membrane can be inhibited by tetherin, which is counteracted by gM. (**D**) Depending on cell type (e.g., polarized epithelial cells), enveloped virions can disseminate through cell-to-cell spread, in a process that requires U_L_7/U_L_51 for sorting of virions towards parts of the membrane that contain gE/gI. Virions may spread to adjacent cells by binding to gE/gI receptors in adjacent cells. However, such receptors are unknown.

VP22 is a 301-aa tegument protein (Figure 3) that is encoded by the U_L_49 gene [188], and has been associated with multiple functions during infection, which result from its interactions with host and cellular factors [189]. It localizes in multiple areas of the cell depending on the time point of the infection, even though its functional role is not always clear. 

Early during infection, VP22 is mostly in the cytoplasm but eventually accumulates in the nucleus. VP22 is phosphorylated after entry into the cell, and this has been suggested to trigger its translocation into the nucleus [190,191]. Inside the nucleus, VP22 may be involved in modulation of nucleosome deposition and repression, which will affect virus life cycle progression [192]. VP22 may also have a function in the nucleolus, since it localizes there during early infection, even though it is not required for chromatin marginalization and HSV-1 replication [193]. 

VP22 associates with ICP0 in the nucleus, and its overexpression affects the transcription of gC and TK1 in the nucleus, suggesting that VP22 affects transcription though an interaction with ICP0 [194]. VP22 regulates the proper subcellular localization of VP16, VP26, ICP0, ICP4, ICP27, and Hsc-70 in infected cells [195]. These proteins localize to the nucleus early during infection and are then translocated to the cytoplasm later in infection. This translocation relies on specific dileucine motifs on VP22 [195]. 

VP22 also seems to be required for the expression of the vhs RNase, suggesting that expression of vhs in the absence of VP22 is lethal [196]. VP22-null mutants accumulate spontaneous secondary mutations in the U_L_41 (vhs) gene, therefore VP22 and vhs may have competitive functions [187]. There is a protein synthesis defect in the absence of VP22, which can result in a compensatory frameshift mutation in vhs. Mechanistically, VP22 and vhs interplay functionally at the level of accumulation and translation of viral mRNAs, indicated by the decrease in mRNA levels and polysome assembly when VP22 is absent. This phenotype can be rescued by the abovementioned complementary mutations in vhs [197]. VP22 is required for optimal protein synthesis at late times during infection, and the accumulation of gE, gD, and vhs mRNAs during early infection [198]. 

VP22 is not required for the accumulation of other tegument proteins, for virion assembly, or productive HSV-1 replication, but the size of the plaques of VP22 mutant HSV-1 strains (lacking the C-terminus) are smaller than the wild type (WT). Therefore, VP22 is probably required for efficient spread [191]. This effect may stem from the multiple interactions of VP22 as a tegument protein at sites of HSV-1 cytoplasmic envelopment, namely the TGN membranes [199]. Optimal packaging of VP22 in virions requires the amino acids 43–86, which facilitate localization of the protein to the TGN [200], but the exact order of VP22 packaging may be flexible. Overexpression of VP22 after infection with a recombinant HSV-1 that has two VP22 copies, resulted in 2–3-fold higher incorporation of VP22 into nascent virions [201]. In any case, the TGN VP22 mediates proper cytoplasmic envelopment as it interacts with the cytoplasmic tail of gD, therefore bridging the viral capsid and the envelope (Figure 3) [202]. Additionally, VP22 interacts with U_L_16 and deletion of U_L_16 results in dramatic reductions of VP22 in released virions [203]. 

Furthermore, it interacts with gE [204,205]. Deletion of VP22 results in reduced amounts of ICP0, gE, and gD in the extracellular infectious virions, whose number is also reduced [206]. Additionally, VP22 bridges a complex between gE/gI and gM, which is selective in its formation, since it does not include VP16, a close partner of VP22 [207]. This VP22/gE/gI/gM complex also recruits ICP0 in a VP22-dependent fashion. None of those proteins is absolutely required for the formation of a subcomplex; however, optimal complex formation results in efficient virus formation (Figure 3) [208]. 

One important aspect of VP22 is its involvement in cytoskeleton reorganization. VP22 requires microtubule reorganization for its translocation to the nucleus [209], and it also affects the reorganization and polymerization of the microtubules late during infection, suggesting a late trafficking role [185]. This occurs independently of virus replication or other viral factors [209,210].

VP22 localization is also affected by motor proteins, like the actin-associated motor protein non-muscle myosin IIA (NMIIA). Inhibition of the ATPase activity of NMIIA impaired the perinuclear vesicular pattern of VP22 and the release of virus into the extracellular space, but it did not affect the cell-associated virus. VP22-containing particles line up along NMIIA-containing filaments that run through protrusions, which can emanate from infected cells [211]. The interactions of VP22 with the cytoskeleton probably affect CD1d-mediated activation of natural killer T (NKT) cells. CD1d is an MHC class I-like molecule that mediates self and microbial lipid presentation to NKT cells. HSV-1 can inhibit CD1d-mediated antigen presentation to NKT cells by suppressing CD1d recycling to the cell surface. VP22 is required for this inhibition of CD1d recycling, which probably occurs because of the reorganization of the cytoskeleton that VP22 promotes, which consequently affects CD1d recycling to the plasma surface [212].

An important host factor that VP22 interacts with is the AIM2 inflammasome, promoting the evasion of AIM2-dependent inflammasome activation during infection. The AIM2 inflammasome is normally activated by DNA, which would be available during HSV-1 infection since the HSV-1 nucleocapsid has been reported to be degraded in the cytoplasm [213]. However, HSV-1 infection induces AIM2-independent inflammasome activation, which is inhibited by VP22. VP22 interacts with AIM2 and prevents its oligomerization, which is the first step in AIM2 inflammasome activation. Mice that lack AIM2 can support infection of a VP22-null HSV-1 [214]. Considering that VP22 can move between cells [215], this would be an efficient manner to block inflammatory responses in uninfected cells adjacent to the infection.

**Figure 4:**
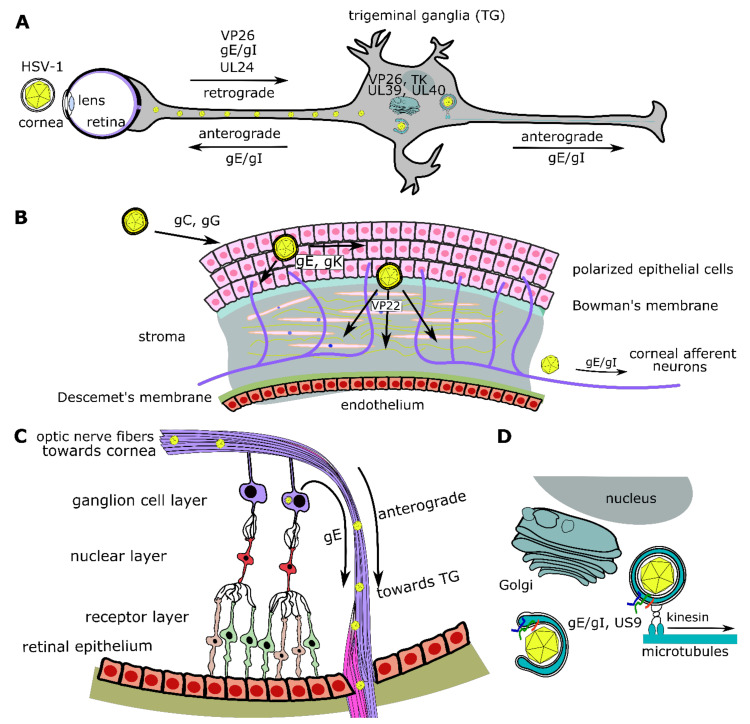
HSV-1 ocular infection based on data in mouse models. (**A**) HSV-1 can infect the eye through the cornea, where it can establish a productive infection in the corneal epithelium. The virus can then spread through the innervating sensory neurons to the trigeminal ganglia (TG), moving retrograde along axons towards the neuronal bodies. VP26 mediates migration to the TG. gE/gI and U_L_24 are important for trafficking of the virion along the axons towards the neuronal bodies. U_L_39/U_L_40 and TK are required for replication in cells that do not actively divide, such as neurons. gE/gI affect both retrograde and anterograde movement of the virion. (**B**) HSV-1 infection of the cornea: The virus can infect the upper layer of polarized epithelial cells, which requires gC and gG. Dissemination of the ocular infection requires the function of gE and gK, and further spread of the virus towards the underlying stromal layers of the cornea requires the function of VP22. HSV-1 can infect corneal afferent neurons and then spread towards the TG utilizing gE/gI. (**C**) HSV-1 can establish an infection in the retinal neuronal cells and can then move retrograde towards the cornea or anterograde towards the central nervous system (CNS). This migration requires gE, and presumably gI since they function in a complex. (**D**) Anterograde trafficking of virions inside neurons requires gE/gI and U_S_9. Based on data in pseudorabies virus (PRV), U_S_9 interacts with kinesins that regulate the anterograde movement of virions along microtubules towards axonal termini. It is also possible that gE/gI and U_S_9 are required for proper cytoplasmic envelopment and sorting to axons.

Another property of VP22 is that it can be transported intercellularly using a Golgi-independent mechanism, which involves the actin cytoskeleton since it is sensitive to cytochalasin D [215]. The importance of VP22 in spread is most evident in animal models. VP22 is required for efficient development of corneal lesions in mice following ocular inoculation (Figure 4) and it is also important for neurovirulence, through two possible mechanisms [206]. First, two dileucine motifs of VP22 (at positions 235–236 aa and 251–252 aa) are required for spread of viral antigens in the mouse brain and efficient virulence. These two motifs have been associated with proper cytoplasmic localization of other viral proteins, and VP22 may mediate neurovirulence though that function [195]. Second, a VP22 mutant HSV-1 exhibits impaired viral replication (about 1000-fold) and spread in the brains of infected mice, supporting the importance of VP22 for virus spread in neurons [195]. VP22 may exert its proviral effect in neuronal spread through blocking of AIM2-dependent inflammasome activation, as explained above. Infection of AIM2^-/-^ mice with a VP22-null HSV-1 results in 3 logs higher viral yield than infection of AIM2^+/+^ mice, suggesting that VP22 promotes neuronal spread by inhibiting an AIM-2-dependent host response against HSV-1 infection [214]. 

The role of VP22 in spread is also used for the transport of viral RNAs during infection to adjacent non-infected cells, but this function can also be utilized for the delivery of products, such as chimeric polypeptides [216]. For example, a chimera consisting of VP22 linked to p53 can spread between cells and accumulate in recipient cell nuclei, while inducing apoptosis in p53-negative osteosarcoma cells [217]. There have been other chimeras that are described in the literature, but the potential of this strategy is not definitive. Not all types of cargo can be carried by VP22, and in vitro data may not translate well in animal models [218,219,220,221,222]. Additionally, VP22 conjugated cargo may be transported, but it might not be functional [223]. These conflicting results suggest that VP22 is not ideal for carrying all proteins, either due to inefficient transport of the chimeras or structural effects of the chimera on VP22. After mapping the functional domains of VP22, engineered versions of VP22 with higher transport efficiency could be investigated [189]. Nonetheless, VP22 chimeras are a promising tool and could be used in cancer, gene therapy, and vaccines. Notably, we have not detected VP22 in CD63^+^ EVs or ESCRT^+^ EVs derived from HSV-1 infected cells, suggesting that the intercellular transfer of VP22 does not depend on light extracellular vesicles (lighter than virions) derived from infected cells [224,225].

A potential avenue for utilizing VP22 transport in cancer therapy involves the introduction into target cells of a nontoxic drug and an enzyme that can convert to it to toxic. For example, introduction in cancer cells of the thymidine kinase (TK) gene and ganciclovir (GCV) results in phosphorylation of GCV, turning it into a nucleoside analogue that kills cells by inhibiting chain extension during deoxyribonucleic acid synthesis. Cytotoxicity is also observed in adjacent cells of a tumor. However, the levels of prodrug that need to be administered to kill adjacent cells in a solid tumor end up being toxic for the patient. These problems of efficient TK and GCV delivery can be resolved through VP22 [226]. VP22 can enhance intercellular trafficking of TK and can amplify the killing effect of the TK/GCV combination, making the fusion of TK and VP22 an attractive candidate for cancer therapy [227]. Another anticancer treatment is the use of the bacterial enzyme cytosine deaminase (CD) with the prodrug 5-fluorocytosine (5-FC), which is converted by CD to the highly toxic 5-fluorouracil. The efficacy of this combination can be enhanced through fusion of CD and VP22. The CD–VP22 fusion has a higher cytotoxicity in mouse models when compared to administration of CD alone [228,229]. 

VP22 could also be used in the context of gene therapy as a carrier [230]. For example, intraocular administration in mice of an adenoviral vector carrying VP22 fused to GFP showed a dramatic increase in the number of CNS neurons expressing GFP versus when administering an adenovirus with just GFP [231]. Other potential uses of VP22 to enhance adenovirus-based gene transfer have been noted in the literature [232]. VP22 can also enhance DNA vaccine protection against *Pseudomonas aeruginosa* in mice [233]. It is possible to fuse VP22 to other antigens of interest inside a DNA vaccine and that can enhance antigen-specific responses and antitumor effects [234].

### 2.4. U_S_1 (ICP22)

The U_S_1 immediate-early gene product, ICP22, is a 420-amino-acid protein. Mutant viruses lacking ICP22 display reduced virus yields in some cell lines, including primary human and rodent cell lines, but not in others, such as Vero (African green monkey) and HEp-2 cells (human epithelial), implying cell type-dependent effects [19,20,235,236,237,238,239,240,241,242]. Using different models of infection in mice and guinea pigs, a virus deleted of ICP22 caused reduced virulence and displayed reduced replication during an acute ocular infection and reduced neurovirulence [238,243,244,245,246]. Homologs of ICP22 are found in other herpesviruses, though the importance of ICP22 in infection seems to differ between viruses [247,248,249,250,251] ICP22 is guanylylated, adenylylated, and is phosphorylated by U_L_13 and U_S_3 [252,253,254,255,256]. Phosphorylation of ICP22 at tyrosine 116 has been found to be important for ocular infection, affecting virulence, but the kinase responsible has not yet been specified [245]. 

ICP22 contains two nuclear import signals and has been implicated in viral gene expression [238,239,257,258]. Particularly, the carboxyl-terminal domain (CTD) of ICP22, in conjunction with the viral U_L_13 protein kinase, was found to enhance the synthesis of a subset of late (γ_2_) proteins exemplified by the products of the U_L_38, U_L_41, and U_S_11 genes (Figure 1C). ICP22 and the U_L_13 protein kinase mediate the activation of cdc2 and degradation of its partners, cyclins A and B. Cdc2 and its new partner, the viral DNA polymerase accessory factor (U_L_42), bind topoisomerase IIα in an ICP22-dependent manner (Figure 1C) [259,260,261,262]. Although topoisomerase II is required for viral DNA synthesis, ICP22 is not, suggesting that the ICP22/topoisomerase II interplay has another role during HSV-1 infection. Indeed, topoisomerase II appears to be required for untangling concatemeric DNA progeny for optimal transcription of late genes. 

Regarding the role of U_L_13 in the abovementioned complex, it was found that ICP22 and U_L_13 are involved in a common pathway that alters RNAP II phosphorylation, and in some cell lines, this change promotes viral late transcription, and also involves U_S_1.5, a shorter gene encoded from the U_S_1 ORF (Figure 1C) [263,264,265,266]. This ICP22/U_L_13-mediated phosphorylation of RNAP II resulted in an “intermediate” electrophoretic mobility between that of hyperphosphorylated (RNAP IIo) and hypophosphorylated (RNAP IIa) states [267]. Furthering this work, it was found that U_L_13 and the C-terminus of ICP22 are both required for RNAP II phosphorylation [267,268,269]. In cells infected with mutants from which U_L_13 had been deleted, ICP22 fails to aggregate in the nuclear structures containing nascent DNA, ICP4, RNA polymerase II, and other factors, implying a role of this U_L_13-mediated phosphorylation in viral late gene expression (Figure 1C) [270,271,272,273]. 

ICP22 was also found to bind the cyclin-dependent kinase 9 (cdk9) but not cdk7, and this complex in conjunction with viral protein kinases (U_L_13 and U_S_3) phosphorylates the carboxyl terminus of RNAP II. The primary function of cdk9 and its partners, the cyclin T variants, is in the elongation of RNA transcripts, although functions related to the initiation and processing of transcripts have also been reported. Cdk9 was found to be important for optimization of the expression of genes regulated by ICP22. Therefore, one function of cdk9 during HSV-1 infection may be to bring ICP22 into the RNAP II transcription complex [274,275,276,277]. In support of these findings, it was reported that ICP22 binds to positive transcription elongation factor b (P-TEFb) and to RNAP II, and along with cdk9 they could suppress the expression of host genes, offering an advantage to the virus [278,279]. In fact, ICP22 represses transcription from all classes of viral genes but selectively upregulates expression of some late (γ2) genes [280,281]. Thus, ICP22 may be important to either repress or activate viral genes at different stages of the viral life cycle.

Besides its functions in viral gene transcription, a regulatory role has been proposed for ICP22 that involves the differential expression of two transcripts produced by the U_S_3 open reading frame. The U_S_3 gene was reported to encode two proteins. In wild-type virus-infected cells, the predominant form is the full-length Us3. However, in ICP22-null virus-infected cells, a shorter form of U_S_3 is produced that initiates from methionine 77 and has been named U_S_3.5 [282]. Like U_S_3, the U_S_3.5 mediates the phosphorylation of HDAC1, HDAC2, the protein kinase A regulatory IIα subunit (PKA RIIα), and the U_L_31 protein. Additionally, both kinases cofractionate with mitochondria. However, the U_S_3.5 failed to block apoptosis (a well-established role of U_S_3) and does not enable efficient release of virus particles from nuclei. Thus, the two proteins differ in the range of functions they exhibit [103].

Another role that has been attributed to ICP22 is involved in the host cell chaperone machinery by facilitating the formation of virus-induced chaperon-enriched (VICE) domains in the nucleus of some infected cells. Recent studies suggested that ICP22 mimics a cellular type II J protein, which is a co-chaperone in the nucleus [283,284,285]. The VICE domains are usually formed adjacent to the viral replication compartments, they contain several host chaperones (Hsp70, Hsp40, Hsp90), proteasomal components, ubiquitinated proteins, and at least one viral protein. These domains are hypothesized to play a role in protein quality control and remodeling, and during infection, they may participate in the formation of viral replication compartments and transcriptional regulation. VICE domains may also allow for correct folding of proteins participating in macromolecular assemblies. VICE domains may have a greater role in certain cell lines, where ICP22 expression is essential [273,286,287,288,289,290].

ICP22 was also found to form a complex with the HSV-1 proteins U_L_31, U_L_34, U_L_47, and U_S_3 (Figure 3). These proteins, as discussed elsewhere, are important for viral egress through the nuclear membrane. ICP22 colocalizes with U_L_31 and U_L_34 at the nuclear membrane in WT virus-infected cells. In U_L_31-null virus-infected cells, targeting of ICP22 to the nuclear membrane is inhibited. In ICP22-null virus-infected cells, U_L_31 and U_L_34 mis-localized in the ER and the nuclear membrane, and significantly reduced the numbers of primary enveloped virions that were observed in the perinuclear space, although capsids accumulated in the nuclei. These data suggest that ICP22 plays a role in HSV-1 primary envelopment by interacting with the nuclear egress complex [291].

More recently, roles for ICP22 in combating the immune system have been proposed. The T cell co-stimulatory molecule CD80 was found to be downregulated in DCs in a manner dependent on ICP22 binding to the CD80 promoter, which seems to limit the pathogenesis of the virus as well as delaying the immune response to infection [292,293,294]. Recent studies with HSV-2 and transfection assays have found that ICP22 may be important in blocking type I IFN responses during infection [295]. Additionally, ICP22 may regulate host E3 ubiquitin ligases. Cumulatively, the findings regarding ICP22 are that it is important for the expression of the late (γ_2_) class of viral genes, formation of viral replication compartment and VICE domains in the nucleus, binding to host transcripts thereby altering host responses to the virus, and even by facilitating nuclear egress of viral capsids.

## 3. Host Evasion Factors

### 3.1. RL1 or γ134.5 (ICP34.5)

The HSV-1 γ_1_34.5 gene product was first described in 1986 [296,297], and HSV-1 and HSV-2 are the only members of the alphaherpesviruses expressing ICP34.5 [298,299]. Deletion of the ICP34.5 gene abolished the capacity of the virus to spread from peripheral mucosal sites to the central nervous system (CNS) or replicate in the CNS, and diminished the capacity of the virus to replicate at mucosal sites and, subsequently, establish latency, or be able to be reactivated ex vivo [300]. In support of this, an ICP34.5-null virus displayed reduced neurovirulence following intracerebral inoculation into mice [298,299,301,302,303,304,305,306,307]. Furthermore, in an ocular model of infection, ICP34.5-null virus did not cause corneal disease [308]. In mouse embryonic dorsal root ganglia (DRG) three-dimensional cultures for HSV-1 latency, a virus with a deletion in ICP34.5 rendered the virus incapable of reactivation, even though the virus was clearly able to replicate and persist in a quiescent form in the DRG neurons [309].

The requirement of ICP34.5 for viral growth is cell type and status dependent, including an inability to replicate in non-dividing cells [303,310,311,312]. The ICP34.5 protein contains a domain homologous to GADD34/MyD116, which functions during growth arrest or DNA damage [313,314,315,316]. In cell culture, this domain of ICP34.5 was shown to be required for preventing the shutoff of host translation through the reversal of phosphorylation of eukaryotic translation initiation factor alpha (eIF2α), which occurs in a PKR-dependent fashion (Figure 2D) [317,318,319,320,321,322]. It was later found that ICP34.5 associates with the host protein phosphatase 1α via its C-terminus and redirects it to dephosphorylate eIF2α (Figure 2D) [317,318,323].

In interferon (IFN)-α/β receptor knockout mice, the ICP34.5-null virus showed a rescue to near wild-type replication levels in the trigeminal ganglia, and this sensitivity to IFN-α/β was found to occur in a manner dependent on the RNA sensor protein kinase R (PKR) [135,324,325]. It was also found that mouse embryonic fibroblasts (MEFs) infected with an ICP34.5-null virus induced higher expression of innate immunity genes and phosphorylation of the transcription factor IRF3, which was partially dependent on TANK-binding kinase 1 (TBK1) binding (Figure 2B) [326]. The binding site of TBK1 to ICP34.5 was found to be dispensable for blocking IRF3 phosphorylation, though ICP34.5 was still demonstrated to be important for this function [327]. 

Another major role for the ICP34.5 protein is blocking autophagy through its interaction with Beclin-1, which has been observed largely in mouse embryonic fibroblasts (MEFs) (Figure 2C) [321,328,329]. Viruses lacking the Beclin-binding domain (BBD) of ICP34.5 were attenuated [328]. Mice infected with the mutant virus missing the BBD of ICP34.5 replicated less in brain and corneal tissue, but the BBD was found to be dispensable for reactivation [330]. The control of autophagy by ICP34.5 was also implicated in preventing MHC-class I antigen presentation of gB from HSV-1-infected macrophages to CD8^+^ T cells through autophagy [331]. In support of this, the BBD of ICP34.5 was found to be important to antagonize autophagy and prevent MHC-class II antigen presentation in dendritic cells (DCs) [332]. Consistently, there seemed to be an increase in CD4^+^ T cell responses, including IFN-γ and IL-2 production in mice infected with HSV-1 lacking the ICP34.5 BBD [330]. However, in MEFs lacking the gene Atg5, which is essential for autophagy, the infection with ICP34.5 deleted for the BBD was not rescued compared to wild-type MEFs and no improvement in virus replication was noticed [333,334]. This suggests that the primary role of ICP34.5 is to counteract PKR activation rather than xenophagy. In support of this, the growth of ICP34.5-deficient virus was completely rescued in PKR^-/-^ MEF cells. The discrepancies in the importance of the BBD and the fact that certain dendritic cell lines and neuroblastoma cells actually exhibiting higher autophagy activation when infected with an ICP34.5-null virus highlight the cell type-dependent role of ICP34.5 [321,328,333,335,336,337]. It was recently described that the expression of the ICP0 protein of HSV-1 was not sustained in the ICP34.5-deleted virus, which complicates our understanding of cellular effects by this virus [327]. More work is needed to clarify how cell type and cell status influences infection with ICP34.5-mutant viruses, and more specifically the ways in which ICP34.5 is able to modulate autophagy in different cells.

### 3.2. U_S_12 (ICP47)

Infection of fibroblasts with HSV-1 renders the cells resistant to lysis by CD8^+^ cytotoxic T lymphocytes (CTLs), which normally recognize cell surface MHC I proteins presenting viral antigens. ICP47 can block the transport of MHC I proteins to the surface, and in this way inhibits lysis of infected cells by CTLs [338]. This explains why using ICP47 in a vaccine vector cannot confer protective immunity in vivo, since it prevents MHC I CTL induction [339]. To prevent the presentation of MHC I molecules on the cell surface, ICP47 binds to the transport-associated with antigen processing (TAP) factor. TAP mediates the transport of peptides destined for presentation by MHC I from the cytosol to the ER [340]. ICP47 binds with high affinity to the substrate-binding site of TAP, preventing the binding of other peptides [341,342], and this binding is species specific, since binding to murine TAP is much weaker [343,344]. This suggests that mice are not the optimal animal model to study the CD8^+^ T cell protective effect of ICP47, but pigs, dogs, or monkeys appear more suitable [345]. Alternatively, recombinant HSV-1 strains that contain murine MHC I complex-binding proteins can be used, and they effectively restrict MHC I antigen presentation in murine models. Work on such models has demonstrated that preventing MHC I antigen presentation increases neurovirulence of HSV-1 since viral entry, replication, and survival in the CNS is possible [346]. 

Preventing MHC I presentation can also be used to enhance the potency of oncolytic mutant strains, such as those based on ICP34.5-null mutants [347,348]. When using oncolytic viruses for cancer immunotherapy, there are issues with the immunogenicity against viral vectors that carry antigens and the memory response that arises after repeated injection of the vector during prime-boost regimens. CTL responses against the vectors prevent build-up of an immune response against the antigen of interest, which in the case of cancer immunotherapy is a peptide that is expressed in tumors. To solve this issue, a tumor peptide can be fused with part of the adenovirus 19K-derived leader sequence (MRYMILGLLALAAVCSA), which is an ER-targeting sequence, so that when expressed in APCs, the fusion product bypasses TAP and traffics to the ER. In the ER, the tumor antigen can be trimmed by aminopeptidases [349], loaded on MHC I molecules, and presented on the cell surface in a TAP-independent manner [350]. In parallel, U_S_12 can be expressed from the viral vector and its expression restricts TAP and TAP-dependent MHC I presentation. In the end, viral vector peptides will not be presented through the TAP pathway and the immunogenicity against the vector will be restricted [351].

This protective role of ICP47 against CD8^+^ T cells is responsible for enhancing HSV-1 neuropathology in vivo. While an ICP47-null virus and WT replicate similarly in corneal epithelial tissues, the ICP47-null virus causes little to no neurologic disease and encephalitis [352]. Mice depleted of T cells can support WT levels of neurovirulence, but mice survival is decreased after exogenous delivery of CD8^+^ T cells [353], suggesting that CTLs can control HSV-1-associated disease. Presentation of HSV-1 peptides to CTLs through TAP is inhibited by ICP47, thus absence of ICP47 restricts neurovirulence whereas absence of TAP does not [354]. Additionally, TAP expression in the brain of infected mice is increased, suggesting that it has a host defense role. Infection with an ICP47-null HSV-1 virus does not trigger an increase of TAP, likely because this virus does not invade the brain [354]. 

## 4. Nucleic Acid Metabolism and Endonucleases

### 4.1. U_L_2, U_L_12, U_L_12.5, U_L_50 

HSV-1 has been shown to encode multiple proteins for the metabolism of nucleic acids in the host cell. One such protein is encoded by U_L_2, and is the uracil DNA glycosylase, which is an important enzyme for removing uracil from DNA [355,356,357,358,359]. The uracil DNA glycosylase activity was described to be important in adult neurons for the replication of the viral genome [360,361]. In support of this, infection of mice with mutant viruses lacking uracil DNA glycosylase activity had significantly reduced viral load in peripheral and central nervous system tissues, as well as reduced reactivation from latency [361]. Later, U_L_2-encoded uracil DNA glycosylase activity was found, with cellular factors and with the viral DNA polymerase, to participate in base excision repair coupled with DNA replication, supporting a role for U_L_2 during genome replication [362,363,364,365]. Moreover, U_L_2 nuclear localization was found to be important for efficient viral replication [366]. U_L_2 was found to be nonessential in cell culture [367]. There is a homolog for this protein in other herpes viruses [368,369,370,371]. 

HSV-1 also encodes an alkaline nuclease or deoxyribonuclease, which is a phosphoprotein with endo- and exonuclease activity, which localizes to the nucleus of infected cells [372,373,374,375]. The alkaline nuclease is encoded by the U_L_12 gene of HSV-1 [22,374,376,377,378,379]. The U_L_12 gene of HSV-1 is highly related in sequence to proteins from other herpes viruses [380,381,382,383]. U_L_12 is not essential for viral DNA replication [384]. U_L_12 is important for viral capsids to egress from the nucleus [385,386]. It was found that cells infected with a mutant virus lacking U_L_12 released many particles with genomes incapable of undergoing new rounds of infection [387]. This increase in defective particles released in the absence of U_L_12 during infection was later found to specifically be due to the nuclease activity of the protein [388].

Mutant viruses lacking alkaline nuclease activity have reduced growth in cell culture, which may be due to a role for U_L_12 in processing viral DNA replication intermediates and packaging the DNA into the capsid [389,390,391]. More specifically, U_L_12 has been found with ICP8, the ssDNA binding protein of the virus, to mediate strand exchange during DNA replication through increased nuclease function by U_L_12 [392,393,394]. U_L_12 also seems to be involved in single-strand annealing during homologous DNA repair in infected cells, which also involves ICP8 as the single-strand annealing protein [395,396]. U_L_12 was also found to interact with components of the MRN (homologous recombination repair complex containing the proteins Mre11, Rad50, and Nbs1) in the nucleus during infection [397]. Together, these reports support a role for U_L_12 in the processing of viral DNA during infection.

The U_L_12.5 protein of HSV-1 has not been fully characterized, but its ORF is known to overlap that of U_L_12. U_L_12.5 lacks the first 126 aa of U_L_12, it retains the nuclease and the ICP8 binding activities of U_L_12, and was initially described as a capsid nuclease [398,399]. However, U_L_12.5 does not accumulate to high levels in the nucleus and cannot efficiently substitute for U_L_12 in promoting viral genome maturation [400]. Interestingly, the only known function of U_L_12.5 seems to be related to mitochondria stress, as it has been found that mitochondrial DNA is eliminated early during HSV-1 infection in a U_L_12.5-dependent manner, which also involves mitochondrial nucleases (Figure 2C) [401,402,403]. This role by U_L_12.5 in altering mitochondria stability during infection has not been further described, but it has been found to be dispensable for viral replication [404]. More work is needed to understand the role of U_L_12.5 during HSV-1 infection.

Another important protein involved in viral DNA replication is the protein encoded by U_L_50 of HSV-1. U_L_50 encodes a tegument protein, which is a deoxyuridine 5′-triphosphate nucleotidohydrolase (dUTPase), which is important for the synthesis of thymidine for DNA replication [153,405,406,407,408]. The dUTPase activity was found to be nonessential in cell culture; however, replication in the CNS of mice infected with a U_L_50-deleted virus was reduced, along with reduced neurovirulence and reactivation [358,409,410]. The dUTPase is phosphorylated by the viral kinase U_S_3 (see below), which seems to regulate the activity of this protein in a cell type-dependent manner, as well as specifically affecting the neurovirulence and replication competency of HSV-1 in the central nervous system [411,412,413]. There are homologs for U_L_50 in other members of the Herpesviridae family [164,380,381,414,415,416,417]. Recent reports studying homologs of the HSV-1 U_L_50 from EBV or using transfections of multiple U_L_50 homologs show the ability of U_L_50 to affect TLR1/TLR2-mediated immune responses and upregulate NF-κB activity [418,419,420]. While it is still not known how this pro-inflammatory effect by the HSV-1 dUTPase affects viral infection or pathogenesis, particularly as it relates to infection of different cell types, it is worth further investigation to understand the immunomodulatory capacity of HSV-1 and the viral dUTPase. 

### 4.2. U_L_39 and U_L_40 (RR1 and RR2)

The holoenzyme of the viral ribonucleotide reductase (RR) is composed of two subunits, the large subunit, also known as ICP6, that is encoded by the U_L_39 gene and the small subunit encoded by the U_L_40 gene. HSV-1 RR converts ribonucleotide diphosphates to corresponding deoxyribonucleotides, allowing for virus replication, particularly in non-dividing cells [421]. Both subunits of the RR are needed for enzyme activity [422], so a decrease in either subunit decreases RR activity. Knockdown of U_L_40 using siRNAs triggers a mild (50%) decrease in plaque size and numbers [423]. Chemical inhibitors of the ribonucleotidase activity can lead to poor viral replication, depending on the cell type [371,424,425]. Dividing cells in S phase contain an elevated dNTPs pool and are capable of supporting replication of ribonucleotide reductase-deficient virus [421,426]. RR is required in non-replicating cells, such as neurons that have a reduced dNTPs pool (Figure 4A). This attribute makes an RR-deficient virus an attractive option for use in oncolytic therapy that targets malignant gliomas, since it generates a non-replicating virus in such tissues, giving a good safety profile [427].

HSV-2 U_L_39 has been associated with antiapoptotic functions [428]. HSV-1 U_L_39 mutants exhibit a 50% reduction in protection from TNFα [429], which suggests that HSV-1 R1 is important for protection of HSV-infected cells from this death ligand, while the 50% efficiency suggests that other viral proteins contribute to this protection. Nonetheless, HSV-2 R1 interacts constitutively with caspase-8 and prevents its interaction with FADD, inhibiting TNFα-mediated apoptosis [430]. 

The large subunit of the RR has been shown to contribute to ocular virulence in mice [431], but null mutants can produce lesions in a guinea pig model [432]. This has also recently been observed with a naturally occurring viral mutant in mice that were impaired in acute replication in the eyes and the trigeminal ganglia of mice, and also defective in establishing a latent infection and reactivation [433]. Interestingly, this mutant cannot inhibit caspase 8-induced apoptosis as wild-type virus [433], further supporting the relevance of the antiapoptotic effects of the RR for pathogenesis. An RR-deficient virus that exhibits impaired acute replication in the eyes and the trigeminal ganglia of mice is an attractive option for a herpes prophylactic vaccine, since it appears to confer protection against HSV-1 challenge post-immunization with an RR-deficient mutant [434]. 

### 4.3. U_L_41 (vhs)

It was first observed that HSV-1 caused the shutoff of host protein synthesis as early as 1978 [435,436,437]. This effect was later ascribed to the U_L_41 gene product of HSV-1, the virion host shutoff (vhs) protein, which is found in the viral tegument [438,439,440,441,442]. There are homologs for vhs in other alphaherpesviruses, such as HSV-2, varicella-zoster virus, equine herpesvirus, and pseudorabies, which is indicative of a conserved benefit for the virus to express this protein [443,444,445]. Although vhs is dispensable in cell culture, its absence in vivo leads to reduced viral pathogenicity and severe attenuation [135,441,446,447,448,449,450]. Vhs was first described to be able to block the accumulation of both host transcripts and all three classes of viral mRNAs [438,439,440,441,451,452,453]. However, it was later suggested that the activity of vhs is regulated late during infection by the viral proteins U_L_47, VP16, and VP22 and this is how it spares beta and gamma viral transcripts, although it downmodulates alpha gene transcripts [187,454,455,456,457]. Vhs blocks the accumulation of mRNAs due to its role as a viral RNase [452,458,459,460]. More specifically, vhs has been identified as an endoribonuclease, with sequence similarities to the FEN-1 family of nucleases, which are found in eukaryotes and archaebacteria and are involved in DNA replication and repair [460,461,462]. Vhs displays substrate specificity similar to that of RNase A and it cleaves at the 3’ end of single-stranded cytidine or uridine residues [463]. A group of mRNAs targeted by vhs includes those with adenylate-uridylate (AU)-rich elements at the 3’ end. Several AU-rich mRNAs are stress response transcripts that are upregulated during HSV-1 infection, as they encode for hostile products, including type-I interferon-related products [457,464,465]. 

These mRNAs appear to be cleaved in ARE and deadenylated in a 3’-5’ decay process occurring in a vhs-dependent manner, whereas the truncated 5’ domains may persist, and this is a mechanism by which HSV-1 counteracts antiviral responses (Figure 2D). Stable host transcripts appear to be targeted by vhs in a different way that includes binding of vhs to the cap structure via its affinity for the translation initiation factor eIF4H that causes mRNA decapping and degradation of the uncapped mRNA 5’ to 3’ [465,466,467]. In addition to eIF4H, vhs also interacts with other subunits of the cap structure, including the eIF4AII isoform, the eIF4B, and perhaps other components of the translation apparatus [460,468,469,470,471,472]. These interactions may allow vhs to access some targeted mRNAs during translation initiation and regulate their expression. Overall, vhs displays specificity since it preferentially degrades translating mRNAs and not tRNAs or rRNAs [473].

By degrading host transcripts, particularly those induced by interferons, vhs has a central role in blocking antiviral responses. It was first described that vhs may be involved in blocking immune responses when it was observed that growth of a vhs-deficient virus was rescued in mice deleted of interferon signaling receptors [135]. It was then shown that there was increased cytokine production in mice infected with a U_L_41-deficient mutant as compared to wild-type virus-infected mice. Additionally, U_L_41 mutant viruses displayed increased sensitivity to interferon-α and -β compared to wild-type virus [474]. Vhs was then described to be important for blocking the activation of dendritic cells (DCs), which was found to occur in a toll-like receptor (TLR)-independent manner [475,476]. It was also demonstrated in mature DCs that vhs is required for HSV-1 to block phosphorylation of STAT1 and IFNγ signaling [477]. Infection of immunocompromised mice lacking the STAT1 gene with a vhs-deficient virus did not rescue the growth of this virus and also resulted in higher induction of cytokines than in wild-type virus-infected mice, indicating that vhs has a fundamental role in promoting virus replication and that STAT1 was required to mount an appropriate non-pathological inflammatory response [478].

In addition, by degrading transcripts, vhs was found to decrease the formation of cytoplasmic stress granules (SGs) in infected cells, thus preventing activation of PKR through accumulation of dsRNAs at the site of SGs, which would otherwise lead to innate immunity activation and translation shutoff due to phosphorylation of eIF2α (Figure 2D) [465,479,480,481,482,483]. Furthermore, vhs has been proposed, with ICP0, to block the DNA sensor IFN-γ-inducible gene IFI16 through its endoribonuclease activity, thus blocking the antiviral activities of IFI16 in multiple cell types [123]. 

Cumulatively, the expression of vhs has been found to benefit the virus in multiple ways, both in vitro and in vivo. 

## 5. Viral Kinases

### 5.1. U_L_23 (TK)

The HSV-1 thymidine kinase (TK) is a 376-aa protein, encoded by U_L_23. TK is responsible for phosphorylating thymidine and deoxycytidine through an ATP-dependent mechanism, though it has been described to have broad substrate specificity [484,485,486,487,488,489]. TK is also known to phosphorylate the nucleoside analogs acyclovir (ACV) and ganciclovir (GCV), which have an inhibitory effect on the viral DNA polymerase, thus blocking viral replication [485,490,491]. TK activity is conserved across other herpesviruses [488,492,493,494,495,496,497,498]. 

In vitro studies have demonstrated that TK is dispensable for virus replication in sensory neurons derived from dorsal root ganglia of rat embryos [499]. However, in vivo studies found that TK is required for virus replication in trigeminal ganglia and the brain but not in peripheral tissues of adult mice (Figure 4A) [500,501,502,503]. This is most likely because adult neurons are post-mitotic and they do not express adequate levels of cellular TK to support the growth of HSV-1 TK-null virus, unlike dividing cells. In support of this, it was shown that substitution of the viral TK with the host TK gene enabled the recombinant virus to replicate in TG [504,505]. In addition, the absence of TK activity impairs HSV-1 reactivation from latency [426,506,507,508,509]. Particularly, it was reported that following corneal inoculation of mice, the HSV-1 TK-null virus was severely impaired for replication in TG [502,510]. However, LAT was expressed in these ganglia, suggesting that the TK-null virus can establish latency [511]. Notably, a TK-null mutant virus cannot reactivate even when latent viral loads were comparable to those that permit efficient reactivation of wild-type virus, indicating that latency establishment was not an issue [237,500,506,508,512,513]. 

Prolonged treatment with acyclovir and its analogs can lead to virus-acquired drug resistance because of mutations accumulating in the TK gene. Such mutant viruses are causing major problems in immunocompromised individuals in the clinic [514,515,516]. Considering also that the properties of HSV-1 TK have been explored in experimental therapies of intracranial tumors, it is important to clarify if HSV-1 TK-null viruses can establish lifelong infections in immunocompromised hosts. Using different HSV-1 TK mutants and different backgrounds of nude mice, it was demonstrated that all HSV-1 TK mutants can establish persistent infections in the TG and brain stem of nude mice [508]. This is consistent with the detection of ACV-resistant TK mutants in the CNS of immunocompromised patients with persistent infection [517].

While the role of TK in viral replication and latency in vivo has been the subject of a fair amount of investigation, a breadth of information has been obtained regarding the potential of TK expression for therapeutic purposes. More specifically, much work has been done on using the HSV-TK/GCV suicide gene therapy system for cancer treatment. This system works such that GCV is monophosphorylated by HSV-TK and further phosphorylated by host cell kinases. The triphosphate form of GCV is an analog of purine and it incorporates in the nascent DNA of the cancer cells, which are actively proliferating and synthesizing DNA. This causes the DNA polymerase to stall with subsequent termination of nuclear and mitochondrial DNA synthesis. As a consequence, DNA damage and cell cycle arrest is induced, leading to caspase-dependent cell death in cancer cells [518,519,520,521,522,523,524]. It was observed in breast cancer cells that the HSV-TK/GCV system induced p53-dependent DNA damage responses and cell cycle arrest, perturbing mitochondrial homeostasis through membrane potential dysfunction and release of cytochrome c into the cytoplasm in neuroblastoma cells [519,525,526]. Similar findings were reported in hepatocellular carcinoma cells using an adenovirus method of delivery for HSV-TK [527]. The HSV-TK/GCV system has also been seen to be effective in tumor models in mice [528,529,530,531]. This system is considered efficient because the effects of HSV-TK/GCV are also mediated through bystander effects on surrounding cells and tissues to those that uptake HSV-TK/GCV, which is thought to occur through the transfer of cytotoxic molecules between cells [532,533,534,535,536,537]. HSV-TK/GCV has since been used in several phase I/II clinical trials [538,539,540,541,542,543]. Other preclinical trials have also been published using HSV TK in other delivery systems, and frequently in combination with other antitumor therapy methods [544,545,546,547,548,549,550].

### 5.2. U_S_3 and U_S_3.5 

The serine/threonine protein kinase U_S_3 of HSV-1 has multiple significant functions, though it is non-essential in cell culture [21]. However, U_S_3 has been found to be critical for infection of both peripheral sites and the central nervous system [237,551,552]. Furthermore, U_S_3 has been implicated in the promotion of the viral infection in multiple ways, from blocking host responses to promoting viral replication and nuclear egress. 

One function of U_S_3 is to promote the egress of nucleocapsids from the nucleus to the cytoplasm through primary envelopment (Figure 3) [553,554]. This is supported by the observation that in U_S_3-null virus-infected cells, aberrant HSV-1 capsids are trapped between the inner and the outer nuclear membrane [103]. Us3 deficiency also causes accumulation of viral proteins essential for cytoplasmic envelopment and viral infectivity, such as gK, with the capsids in the perinuclear space (PNS) [555]. To further promote nucleocapsids egress, U_S_3 has been implicated in phosphorylation of lamin A/C that causes changes in its architecture, including changes in its localization and conformation [556,557,558,559]. U_S_3 also assists in the egress of nucleocapsids through interactions with and phosphorylation of U_L_31 [560] and U_L_34 [556,561,562]. All three proteins are located within perinuclear virions and at the inner nuclear membrane (INM), suggesting that they could be incorporated into the virion during budding at the INM. U_S_3 also phosphorylates U_L_47 at Ser-77, promoting its nuclear localization [563]. A proposed role of nuclear U_L_47 is to interact with the nuclear egress factors U_L_31, U_L_34, and U_S_3 to regulate viral nuclear egress [291].

Another function of U_S_3 is inhibition of apoptosis [564,565]. It has been demonstrated that expression of U_S_3 protein outside of the context of the infection mediated post-translational modification of BAD, a proapoptotic protein, which is no longer proapoptotic upon post-translational modifications at Ser-112 and Ser-136 by U_S_3 [566]. Consequently, PARP cleavage, BAD cleavage, and caspase 3 activity were blocked when proteins that induce apoptosis were expressed concordantly with U_S_3 [470,566,567]. Us3 may block apoptosis in multiple ways since it blocks cell death induced after infection with various HSV-1 mutants, such as ΔICP4, but it can also protect against cell death induced after thermal or osmotic shock [564,568]. In support of this, the optimal consensus sequence of U_S_3 peptide substrates was found to resemble the target sequence of the cellular cAMP-dependent protein kinase PKA [569]. PKA is a key enzyme important in the regulation of metabolism, survival, and proliferation of eukaryotic cells, and it mediates most of the biological effects of the second messenger cAMP. The pattern of proteins phosphorylated by U_S_3 overlaps that of phosphoproteins targeted by PKA. Consistently, PKA could block apoptosis by stimuli that Us3 could block. Overall, U_S_3 can block DNA fragmentation and cell death caused by exogenous expression of pro-apoptotic factors or a variety of other stimuli. 

U_S_3 has been implicated in promoting viral gene expression both at the level of transcription and translation and virus replication (Figure 1). U_S_3 promotes viral gene transcription by preventing the deacetylation of histones, a function that also involves ICP0 [103,570]. The U_S_3 kinase from VZV and PRV promotes hyperphosphorylation of HDAC2 and likely HDAC1 to reduce viral genome silencing and allow efficient viral replication [570]. U_S_3 has also been proposed to act as the ser/thr kinase Akt, although it does not look like Akt. This Akt-like kinase function of U_S_3 has a role in stimulating mRNA translation through activation of mTORC1 by phosphorylating tuberous sclerosis complex 2 (TSC2) on the same sites as Akt [571,572]. Activated mTORC1 negatively regulates the activity of the translation repressor 4E-BP1, enabling cap-dependent translation. Additionally, U_S_3 phosphorylates the viral dUTPase, encoded by U_L_50, at Ser-187, and that causes an increase of its activity over host dUTPases, thereby promoting HSV-1 replication [411]. 

Finally, U_S_3 is implicated in virus defense against the host. One example is the requirement of U_S_3 for inactivation of CD8^+^ cytotoxic T lymphocytes, thus preventing cytokine production [573]. U_S_3 also blocks TLR2 signaling early during infection by preventing TRAF6 polyubiquitination [574]. Additionally, U_S_3 hyper-phosphorylates IRF3 at Ser175, which inhibits IFN-β production [575]. Furthermore, the Bcl-2-associated transcription factor 1 (Bclaf1) was recently shown to be degraded in a U_S_3-dependent manner during HSV-1 infection [576]. This degradation prevented IFN-α-mediated interferon-stimulated genes expression [576]. U_S_3 also phosphorylates both ULK1 and Beclin-1, thus blocking autophagy activation during HSV-1 infection in a manner independent of ICP34.5 [577]. In a different approach to promoting the viral infection, U_S_3 has also been found to affect the subcellular localization of certain viral proteins by affecting their phosphorylation status. Particularly, U_S_3 causes a decrease in the amount of gB found on the cell surface by phosphorylating its cytoplasmic tail at Thr-887 and perhaps enhancing its endocytosis [578]. This phosphorylation of gB has been implicated in virus pathogenesis since mutation of Thr-887 significantly impaired viral replication in the mouse cornea and the development of herpes stromal keratitis and periocular skin disease [578]. U_S_3 has also been found, with gB, to be involved in downregulation of the major histocompatibility complex class I-like antigen-presenting molecule, CD1d, through prevention of recycling of CD1d to the cell surface [579]. 

A shorter version of Us3 named Us3.5 was discussed earlier together with ICP22.

Overall, U_S_3 plays many important roles in the viral life cycle and in viral infectivity.

### 5.3. U_L_13 

In addition to the U_S_3 kinase, HSV-1 encodes a second serine/threonine protein kinase U_L_13, which functions in the cell nuclei and is present in the virion as a tegument protein associated with the capsid [580,581,582,583,584]. U_L_13 is conserved across members of alpha-, beta-, and gamma-herpesviruses [380,585,586,587,588,589]. U_L_13 is non-essential in cell culture, but it seems to have a role in counteracting antiviral responses and is important for optimal viral replication in cell culture [590,591,592]. In a mouse model, a U_L_13-deleted virus was sensitive to type I IFN, suggesting an important role for U_L_13 in blocking host responses to infection [593].

U_L_13 phosphorylates multiple viral proteins, including itself, the immediate-early viral protein ICP22 and U_S_1.5, ICP0, and numerous tegument and envelope proteins (Figure 1) [190,242,253,254,592,594,595]. It seems that phosphorylation of ICP0 by U_L_13 is important for stabilizing ICP0 protein during infection [596]. This is supported by the fact that ICP0 is degraded both early and late in cells infected with a mutant lacking the U_L_13 protein kinase. Furthermore, it was found that ICP0 encoded by wild-type virus or the U_L_13-null mutant is stable in cells transfected with a plasmid encoding U_L_13 before infection [596]. Phosphorylation of U_L_46 by U_L_13 leads to Akt activation to promote cell survival [597]. U_L_13 also phosphorylates VP22 at casein kinase II consensus sites, but it was found that U_L_13 modulates cellular localization of VP22 in a phosphorylation-independent manner [590,595,598]. The significance of phosphorylation of VP22 by U_L_13 has not been investigated. U_L_13 phosphorylates the viral Fc receptor gE/gI, though it seems that a host kinase may also phosphorylate gE/gI [599]. Phosphorylation of gE by U_L_13 is thought to facilitate packaging of U_L_13 into the virion [599]. However, this phosphorylation could also impact gE trafficking or other functions. U_L_13 expression was found to be downregulated by the U_S_11 RNA-binding protein. U_S_11 was found to bind to the RNA sequence, designated as 12/14, which is present in the coterminal HSV-1 mRNAs U_L_12, U_L_13, and U_L_14. This binding led to reduced U_L_13 kinase activity due to reduced mRNA levels [600]. While the exact mechanism of downregulation of U_L_13 transcripts by U_S_11 is unknown, it is thought that this occurs through nucleocytoplasmic export of the transcript [600]. The kinase activity of U_L_13 and U_S_3 was found to be important for the viral glycoproteins gC and gD to be modified and expressed late during infection, as loss of both U_L_13 and U_S_3 diminished virion release, showing a role for U_L_13 in assembly and egress of the virion [601]. Thus, U_L_13 modifies multiple viral proteins both to promote assembly and release of the virus, and to affect the stability and localization of viral proteins.

U_L_13 plays roles in modulating host responses to infection. For example, interferon stimulation and production of cytokines are modulated by U_L_13 during infection. U_L_13 has been found to be important for the induction of a set of suppression of cytokine signaling (SOCS) genes late during infection, which is important for blocking the interferon response during HSV-1 infection of cells [602,603,604,605]. Recently, U_L_13 was found with the other viral kinase U_S_3 to be important for regulating phosphorylation of protein kinase R (a nucleic acid sensor) during infection [606]. U_L_13 was also found to hyperphosphorylate an important host factor for the elongation of peptide chains during mRNA translation, eF-1δ during HSV-1 infection, supporting viral protein synthesis, and also preventing apoptosis [607,608,609]. U_L_13 was found to phosphorylate the cellular casein kinase II β subunit (CKIIβ), though the significance of this phosphorylation has not been explored [610]. However, it was found that U_L_13 is able to phosphorylate proteins at similar residues as the cellular cdc2 cyclin kinase [610]. It was also determined that U_L_13, with ICP22, is responsible for activating cdc2 during infection, which is required for optimal expression of viral late genes [259,260]. U_L_13 was also found with ICP22 to phosphorylate the RNA polymerase II (RNAP II), supporting virus late gene expression [267]. Overall, phosphorylation of host proteins by U_L_13 supports viral infection by supporting virus late gene expression, blocking innate immune responses, supporting viral protein synthesis, and activating cellular proteins for the benefit of the virus.

## 6. Virion Morphogenesis, Egress, Cell-to-Cell Spread, and Host Evasion

### 6.1. U_L_3 and U_L_4

The HSV-1 U_L_3 and U_L_4 are late proteins that have only minorly been studied, though they have been determined to be nonessential in cell culture and U_L_4 was also found to be nonessential in mouse models of HSV-1 infection for latency, reactivation, and pathogenesis [421,611,612]. The U_L_3 phosphoprotein and the U_L_4 protein have homologs in other members of the *Herpesviridae* family [84,153,368,369,370,613,614,615,616,617,618,619,620]. U_L_3 appears to localize perinuclearly early during infection and in nuclear puncta at late times post infection [613,621,622]. U_L_3 and U_L_4 have also been found in the nuclei of infected cells and found to co-localize with ICP22 in nuclear bodies, which may involve recruitment by ICP22 [271,623,624,625].

### 6.2. U_L_7 and U_L_51

HSV-1 pU_L_7 is a 296-aa tegument protein [626] that lacks a putative transmembrane sequence or motifs that could facilitate its membrane anchor. Therefore, its association with membranes is mediated through the interaction with a membrane protein, i.e., U_L_51. U_L_7 forms a complex with U_L_51, and this is required for the recruitment of U_L_7 to cytoplasmic membranes and into the virion tegument (Figure 3C) [627]. pU_L_7 and pU_L_51 form a stable and direct protein-to-protein interaction [628], and they function as a complex in infected cells. Both are important for HSV-1 assembly and plaque formation. Their individual ablation results in similarly lower yields with a double U_L_7/U_L_51 knockdown, suggesting that U_L_7 and U_L_51 work in the same pathway [628], which is likely related to cytoplasmic envelopment of HSV-1 virions since many unenveloped capsids next to membranes can be seen in cells infected with U_L_7/U_L_51 mutants [628]. 

U_L_7 ablation can affect HSV-1 infection at earlier times, too. U_L_7 absence results in viruses with lower yields in vitro and lower pathogenic effects in vivo [629]. Mice infected with a U_L_7 mutant HSV-1 exhibit longer survival than those infected with WT HSV-1. A decrease in LAT mRNA expression was observed in the CNS and trigeminal ganglia of mice infected with a U_L_7 mutant HSV-1, suggesting decreased viral gene transcription in the absence of U_L_7. This is supported by data that show that U_L_7 may participate in the complex that is involved in the transcription of ICP4 [629]. It is not clear if U_L_7 is directly involved in the interaction between the promoter of ICP4 and the transcriptional complex or is involved perhaps in the chromatin remodeling process that enables transcription of ICP4. U_L_7 can be detected through cell fractionation and fluorescence microscopy in the nucleus [630], although it is primarily seen in the cytoplasm. It is not clear if its function in the nucleus is derived from U_L_7 delivered as part of the incoming virions [627] or from nascently expressed U_L_7. 

U_L_7 and U_L_51 can also affect cell-to-cell spread of HSV-1. pUL51 interacts with pU_L_7 and gE/gI in infected cells, and deletion of part of U_L_51 or deletion of U_L_7 results in failure of gE to concentrate at junctional surfaces of Vero cells (Figure 3). This suggests a role for a U_L_51/U_L_7 complex in cell-to-cell spread of HSV-1; however, this may depend on the cell line. A pU_L_7/pU_L_51/gE/gI method for cell-to-cell spread can occur in polarized epithelial cells, such as HaCaT, but different cell-to-cell spread mechanisms may be utilized in non-polarized cells, such as Vero [631]. Additionally, the U_L_7/U_L_51 complex can affect cell-to-cell spread by localizing to focal adhesions in infected cells. Focal adhesions are contact sites between the cytoplasm and the extracellular matrix. They are dynamic and respond to extracellular stimuli and play a role in cell attachment and movement. Ablation of the U_L_7/U_L_51 complex results in destabilization of focal adhesions and diminished cell integrity [628]. U_L_7 and U_L_51 seem to mediate the stability of focal adhesions during infection, perhaps to maintain the proximity of infected and non-infected cells so that cell-to-cell spread can be promoted. This still remains to be demonstrated.

An additional function of U_L_7 may be exhibited on the mitochondria. U_L_7 has been identified as a partner of the adenine nucleotide translocase (ANT2) through a mass spectrometry approach combined with affinity purification. ANT2 localizes in the inner mitochondrial membrane and is a member of the permeability transposition pore complex. In physiological conditions, it exchanges ATP and ADP on the inner mitochondrial membrane and is essential for maintaining the cell metabolism exchange of cytosolic ADP for mitochondrial ATP. HSV-1 infection can affect mitochondrial function, and U_L_7 may be one of the proteins that are important for this function [632].

U_L_51 is a late gene that is expressed as three phosphoproteins with sizes of 27, 29, and 30 kDa. It can be detected in extracellular HSV-1 virions [633], is phosphorylated on five sites [634], and phosphorylation on the Ser-184 site has been described as important for HSV-1 replication in vitro and pathogenicity in vivo after ocular infection of mice [634]. U_L_51 localizes in the cytoplasm [633], mostly in the perinuclear area, but part of it also localizes to the Golgi. Golgi localization requires the N-terminus of U_L_51, which is palmitoylated to mediate sorting to Golgi membranes. U_L_51 is packaged in virions, on the inside of the viral envelope [633]. U_L_51 internalization into vesicles and virions may occur during cytoplasmic envelopment in infected cells [633]. Infections with a U_L_51-null HSV-1 yields smaller plaques and a growth of 2 logs lower than a WT HSV-1. U_L_51-null infections exhibit enveloped virions at the perinuclear space, as opposed to enveloped virions at membranes at the TGN during WT HSV-1 infections [635]. The membranes that encapsulate those U_L_51-null virions resemble nuclear membranes through electron microscopy. Such membranes are tightly wrapped around nucleocapsids and do not appear as fuzzy as the membrane of the extracellular virions. Additionally, nucleocapsids are found intranuclearly adjacent to the inner nuclear membrane (INM) with membranes with the same appearance, further supporting that these perinuclear enveloped virions are enveloped with membranes derived from the nuclear cisternae. This suggests that U_L_51 acts at a post-inner nuclear membrane envelopment step, possibly during the outer nuclear membrane de-envelopment process (Figure 3B).

As mentioned above, U_L_51 has a role in cell-to-cell spread that is dependent on cell type. U_L_51 colocalizes with gE in infected cells and it can be immunoprecipitated together with gE, in addition to affecting gE localization to cell junctions [636]. It is possible that U_L_51 functions as a trafficking mediator while is present on the cytoplasmic side of Golgi membranes.

U_L_51 recruits U_L_7 into the nascent virion tegument (Figure 3) [627]. Their colocalization is incomplete though, suggesting that they have other independent functions [627]. 

U_L_51 also interacts with U_L_14 in infected cells, as shown by affinity purification [637]. Three amino acids on U_L_51 are required for this interaction, and their mutation results in decreased viral replication and accumulation of unenveloped and partially enveloped capsids in the cytoplasm. The localization of both U_L_51 and U_L_14 depends on their reciprocal interaction. These data suggest the U_L_51-U_L_14 complex regulates cytoplasmic envelopment of HSV-1 [637]. 

### 6.3. U_L_10 and U_L_49.5 (gM and gN)

Glycoprotein M (gM) is an integral viral envelope membrane protein that spans the membrane eight times [638]. Its deletion results in only a small decrease in viral yields in cell culture [611,639], thus it is defined as non-essential. Even though a gM-null virus can establish a latent infection in mice, it is impaired for growth within the nervous system versus the wild-type virus [638].

gM localizes within the leaflets of the nuclear membrane, at the Golgi and the TGN, and the envelopes of cytoplasmic and extracellular virus particles [640]. Infection with a U_S_3-null HSV-1 results in punctate extensions and invaginations of the nuclear membrane, on which gM localizes [640]. This suggests that gM becomes incorporated into the virion envelope upon budding through the nuclear membrane. Transfection of gM leads to its localization to the TGN and plasma membrane (PM) [641,642]; however, this pattern changes during infection. When gM is expressed, it is recruited to nuclear membranes and then to perinuclear virions once they are formed. This occurs before HSV-1 induces reorganization of the TGN and before gM localization to the TGN. Consistent with these observations, confirmed partners of gM, such as gH/gL, gN, VP22, U_L_31, and U_L_34 [643,644,645,646], do not colocalize with gM early during infection. Therefore, it has been proposed that the function of gM early during infection in the nuclear membranes is separate from its function during viral egress [641]. Characterization of gM domains showed that its trafficking to the TGN requires its transmembrane domains, while its C-terminal trafficking motifs are dispensable. The requirement of the transmembrane domains suggests that gM may associate with other transmembrane proteins for trafficking (Figure 3C) [647], but this remains to be shown.

The role of gM in the TGN is related to the trafficking of host and viral proteins during infection. For example, co-expression of gB, gD, gH, and gL can trigger fusion of cell membranes of transfected cells. Such fusion can be inhibited by the additional co-expression of gM [643]. In this context, gD and gH/gL can be seen to relocalize from the plasma membrane to the TGN, suggesting that inhibition of fusion is triggered through the removal of the fusion glycoproteins from the surface [643]. These data suggest that gM is involved in either retaining viral glycoproteins at the TGN or causing their translocalization from the plasma membrane to the TGN, supporting virion maturation at the TGN [648]. Further supporting data show that an absence of gM results in reduced gH/gL internalization from the PM of infected cells, and reduced incorporation in produced virions [649]. 

gM can interact with gN, resulting in altered intracellular targeting of both proteins. Co-immunoprecipitations in transfected or infected cells indicate that gM and gN form a complex [642], and gN overexpression seems to mediate the formation of syncytia in infected cells, which are inhibited normally by gM [642,643,650]. Syncytia occurs when cell membranes fuse, forming large multinucleated cells. This suggests a strict regulation of fusion that can be deregulated by altered gN expression possibly through altering the localization of gD and gH/gL from the plasma membrane to the TGN that is triggered by gM [643]. gN is an ER-resident protein that in the presence of gM is translocated to the TGN. gM and gN are covalently linked between two cysteines, and exit of gN from the ER requires the N-terminus of gM but not the C-terminus. gN is non-essential and its deletion does not seem to affect viral growth [644].

While gM can inhibit syncytium formation in transfected cells [643] and reduces the surface expression of proteins involved in fusion, only gN and U_L_46 have been identified as partners of HSV-1 gM. Proteomics studies with an emphasis on host proteins identified the host extended synaptotagmin 1 (E-Syt1) as a gM partner [651]. E-Syt proteins promote the close apposition of the ER and the plasma membrane (PM), and the transfer of lipids between the ER and the PM. Functions of several synaptotagmins remain to be determined, but they seem to engage and regulate SNARE proteins (the core cellular fusion machinery) and act as Ca^2+^ sensors. 

It was found that during HSV-1 infection, knocking down E-Syt1 triggered the release of the virus into the extracellular space, at the expense of cell-associated infectious particles. Conversely, overexpressing E-Syt1 led to reduced levels of mature virions in the medium, hinting at a negative regulation. E-Syt1 did not act alone but in combination with the related E-Syt3, which exhibited a similar phenotype. Most interestingly, these E-Syt proteins impacted viral entry, as well as cell–cell fusion (syncytia) and viral plaque size (cell-to-cell spread), suggesting they acted on the viral fusion machinery [651]. One possible mechanism of action might involve deregulation of Ca^2+^ signaling that occurs during HSV-1 infection [652,653], and which could affect E-Syt Ca^2+^-dependent function [651]. 

A BioID proteomics approach identified multiple gM partners, with 35% of those being involved in protein transport. XPO6, an exportin, is required for gM to be released from the nucleus to the TGN [654].

Another interesting way that gM is involved in affecting host trafficking is through modulation of tetherin (Figure 3). Tetherin is an effective cellular factor against a variety of enveloped viruses. Its antiviral activity stems from its ability to form a tether between a host membrane and a budding viral envelope, inhibiting the release of budding virions [655]. Tetherin can also target HSV-1, as the overexpression of tetherin led to accumulation of HSV-1 particles to the cell surface, suggesting inhibition of HSV-1 release [656]. HSV-1 counteracts tetherin function through gM, which interacts with tetherin and removes it from the plasma membrane, thus preventing virion tethering to the plasma membrane. This antagonistic effect might be due to gM preventing tetherin reaching the cell surface or relocalizing tetherin away from the plasma membrane [656], perhaps in a similar manner with gD and gH/Gl [643].

### 6.4. U_L_11

U_L_11 is an early expressed 96-aa myristylated and palmitoylated tegument protein [657,658,659] that is not required for HSV-1 replication in cell culture. A U_L_11-null virus exhibits smaller plaques and displays about one log decrease in progeny virus production [660]. Additionally, the palmitoylation and myristylation of U_L_11 is not required for viral growth, since a non-myristylated U_L_11 mutant HSV-1 can rescue the growth of a U_L_11-null HSV-1 [661]. The palmitoylation of U_L_11 is required though for association of U_L_11 with the cytoplasmic faces of Golgi membranes of infected cells [658,660]. 

U_L_11 can interact with several viral proteins, including U_L_16, as observed through immunoprecipitation, mass spectrometry, and yeast-two-hybrid assays [155,662]. There are dileucine and acidic cluster motifs on U_L_11 that are required for the U_L_11–U_L_16 interaction [659], as well as the free cysteines of U_L_16 [663].

It is not clear what the mechanism of the packaging of U_L_11 in the tegument of nascent virions is [659]. Tandem affinity purification (TAP) supports that U_L_11 interacts specifically with the cytoplasmic domain of gD and gE [664]. In the absence of the cytoplasmic tail of gE, virion packaging of U_L_11 was reduced by 80% [665]. Similarly, gE packaging is reduced 85% in the absence of U_L_11, as gE packaging requires the U_L_11 acidic cluster [665]. These data highlight the importance of U_L_11 in recruiting glycoprotein-enriched membranes for cytoplasmic envelopment of the virus and could have implications for gE/gI-mediated cell-to-cell spread of HSV-1 (Figure 3C,D) [665]. Interestingly, deletion of the gD cytoplasmic domain still allows partial binding of U_L_11 to the ectodomain of gD, suggesting that U_L_11 can be highly adherent (“sticky”) [664]. This would support that adherent tegument proteins can support extensive protein–protein interactions, which would mediate the bridging of the viral capsid to the envelope. U_L_11-null virus infections result in accumulation of unenveloped capsids in the cytoplasm surrounded by electron-dense material (most likely other tegument proteins) [666]. 

The “stickiness” of U_L_11 can be explained by its description as an intrinsically disordered protein (IDP). IDPs contain amino acids and elements that cause them to exhibit hallmarks of a disordered structure, such as slower electrophoretic mobility than expected based on length, reduced size exclusion chromatography mobility due to reduced protein compaction, and low proportion of hydrophobic amino acids with a high proportion of charged and hydrophilic amino acids. The result is a protein that cannot fold spontaneously into a stable conformation and fluctuates rapidly through a range of conformations. Such proteins frequently interact and function within protein–protein interaction networks [667]. As a result, U_L_11 can undergo phase separation in vitro and form biomolecular membrane-less condensates. Such condensates can contain one or many different kinds of proteins. Specific conditions may mediate the formation of condensates by U_L_11 in cells, such as binding to gE, U_L_16, or clustering within lipid rafts. Other HSV-1 tegument proteins also have IDP regions, indicating that phase separation may be used for tegument packaging during HSV-1 cytoplasmic envelopment [668]. 

If one deletes the dileucine and acidic cluster (AC) motifs of U_L_11, then U_L_11 increases its association with detergent-resistant membranes (DRMs), which are enriched in cholesterol and sphingolipids. One possibility is that the deletion of dileucine motifs and ACs results in palmitoylation and myristylation to recruit U_L_11 to DRMs [669]. 

### 6.5. U_L_16

U_L_16 is an unusual gene because it is contained within the intron of the U_L_15 gene and is transcribed antisense to the U_L_15 gene [611]. U_L_16 has a bewildering number of interactions with gE, U_L_11, U_L_21, VP22 (Figure 3) [203], gD, and mitochondria [670]. These interactions are probably regulated temporally but also structurally by different domains of U_L_16.

U_L_16 resides on cytoplasmic capsids [671] and participates in a bridging interaction with membrane-bound U_L_11 [203,662]. This suggests a role for U_L_16 in HSV-1 cytoplasmic envelopment, which became clear in electron microscopy studies of cells infected with U_L_16-null HSV-1 [203]. No defects in the transport of capsids to cytoplasmic membranes were observed, but the wrapping of capsids with membranes was delayed. Moreover, clusters of cytoplasmic capsids were observed but only near membranes where they were wrapped, resulting in multiple capsids within a single envelope. Post-envelopment egress does not require U_L_16, and viruses released in the supernatant were not affected by U_L_16 ablation [203]. Additionally, less gE and less gD were packaged in U_L_16-null viruses, which is expected since U_L_16 interacts with both [672]. These data support a role for U_L_16 in cytoplasmic envelopment. Cell-to-cell spread is also blocked during U_L_16-null virus infection, which may be due to mislocalization of gE, since gE and U_L_16 form a complex [672]. 

The structural modulation of U_L_16 interactions became clear in the studies of the U_L_16/U_L_11/U_L_21/gE complex. U_L_16 directly interacts with U_L_11, which resides on the cytoplasmic side of the TGN. This interaction requires most of the U_L_16 sequence except the first 40 aa, and does so in a manner that requires free cysteines on U_L_16 [663]. Covalent modification of the U_L_16 free cysteines with N-ethylmaleimide blocks binding to U_L_11 but not to U_L_21 [673], suggesting a binding site on U_L_16 for U_L_11 and another for U_L_21 (Figure 3C). 

Interestingly, U_L_16 is released from capsids upon binding of HSV-1 virions to cells, but it is not clear if it maintains its interaction with U_L_11 [674]. For U_L_16 to receive a signal from outside the virion, it must interface in some manner with glycoproteins on the surface of the virion. One possibility is through gE, with which U_L_16 interacts, as discussed above. The N-terminus of U_L_16 can bind gE but the full length cannot, indicating a possible regulatory effect of the U_L_16 C-terminus on the U_L_16–gE interaction [675]. This interaction may have multiple effects, including effects on cytoplasmic envelopment described above. Additionally, since gE is involved in cell-to-cell spread, it may be involved in rearrangements that occur upon binding of virions to cell entry receptors, which results in release of U_L_16 from the viral capsid [663]. 

The inconsistency between the N-terminus and the full-length binding of U_L_16 with gE became clearer when it was shown that U_L_21 binding to U_L_16 reveals the U_L_11-binding free cysteine-requiring site of U_L_16. Then, U_L_11 binds to U_L_16 and this event activates the U_L_16–gE interaction. Importantly, the function of gE is dependent on U_L_11, U_L_16, and U_L_21, as evidenced by infections with HSV-1 gBsyn mutants that lack U_L_11, U_L_16, or U_L_21. The syncytial phenotype of gBsyn HSV-1 infections requires functional gE, and syncytia cannot form in the absence of U_L_11, U_L_16, and U_L_21. Cell-to-cell spread involves the localization of gE to junctions at the cell surface, and in the absence of U_L_11, U_L_16, or U_L_21, gE cannot localize there. Collectively, these data suggest that these proteins work as a complex during HSV-1 infection [676].

One interesting aspect is the species-specific requirement for the U_L_16 protein when comparing HSV-1 and HSV-2 [677]. Depletion of U_L_16 in HSV-2 results in 50- to 100-fold lower viral yields, with defects in both nuclear egress and cytoplasmic envelopment. In contrast, depletion of U_L_16 in HSV-1 results in a 10-fold replication deficiency and defects in cytoplasmic envelopment of viral capsids. HSV-1 U_L_16 can promote the nuclear egress of HSV-2 U_L_16-null mutants, suggesting that HSV-2 lacks an activity that can promote nuclear egress in the absence of U_L_16, as opposed to HSV-1 [677].

A U_L_16-null virus is greatly diminished in its ability to package gD. U_L_16 binds directly to the cytoplasmic tail of gD. If the cytoplasmic tail of gD is removed, U_L_16 is still packaged into virions [678]. This non-reciprocal interaction suggests that packaging of U_L_16 on capsids is independent of gD, but recruitment of gD during cytoplasmic envelopment may require U_L_16. 

### 6.6. U_L_53 (gK) and U_L_20

Glycoprotein K (gK) is a highly hydrophobic 338-amino-acid protein that is encoded by the U_L_53 gene [679]. gK is highly embedded on the membrane [680], which makes its study difficult. Its structure is composed of three or four transmembrane domains, as shown by tag insertions in multiple domains of gK [680]. 

Deletion of HSV-1 gK results in a small plaque phenotype, lower viral yields, and accumulation of non-enveloped virion particles in the perinuclear space [681,682]. These data suggest that gK has a role in the egress of virus from infected cells (Figure 3). Further work demonstrated that deletion of gK triggers a collapse of the Golgi to the ER, in a manner similar to brefeldin A [680]. Virion entrapment in this perinuclear space may occur due to this Golgi collapse, and it has been suggested that this collapse may be partially due to an antifusogenic role of gK during egress. 

The antifusogenic role of gK has been studied extensively. Cell-to-cell transmission of HSV-1 occurs by either release of virions to the extracellular space or virus-induced cell-to-cell fusion. Certain spontaneous mutants of HSV-1 have been found to induce the formation of large multinucleated cells or syncytia. Such mutations have been identified in gK and in other viral glycoproteins, such as gB, but those in gK are more frequently observed [683]. The syncytial gK mutants of HSV-1 have been used in multiple studies to investigate the function of gK during infection.

When gK is expressed outside the context of the infection, it localizes in the ER and the perinuclear space. However, infection of syncytial gK-transfected cells with a gK-null virus triggered expression of gK on the cell surface and cell fusion [680]. Wild-type gK can also inhibit fusion that is triggered by other HSV-1 glycoproteins outside the context of the infection. Co-transfection of the four viral glycoproteins gD, gB, gH, and gL triggers cell-to-cell fusion and leads to syncytia. However, co-expression of wild-type gK with these glycoproteins reduces dramatically the formation of syncytia [684]. Therefore, it was suggested that gK is part of the mechanism through which HSV-1 regulates its own fusogenic activity. The anti-fusogenic activity of gK might prevent fusion of the viral envelope with the membrane of exocytic vesicles as the virus leaves the cell. In a similar manner, it could prevent the collapse of Golgi to the ER that was described above. BFA works by blocking vesicle and protein transport from the ER to the Golgi, and gB could affect Golgi integrity by blocking vesicles feeding into the Golgi from the ER [680].

The antifusogenic role of gK can also be seen during the absence of U_L_20 in U_L_20-null virus-infected cells. This virus produced smaller plaques, and electron microscopy of infected cells showed accumulated capsids in the cytoplasm with few enveloped virions inside cytoplasmic vesicles [685]. A gK syncytial mutant in a U_L_20-null genetic background did not allow cell fusion as seen previously. Additionally, multiple virion capsids within a single envelope were seen in the cytoplasm, further supporting the anti-fusogenic role of gK during viral egress and also indicating an indirect role for U_L_20 in membrane fusion [685].

The U_L_20 gene encodes a 222-amino-acid non-glycosylated transmembrane protein that is conserved in all herpesviruses. It was thought that U_L_20 was essential for virus replication since deleting U_L_20 prevented replication. However, experiments utilizing 143TK^-^ cell lines indicated cell type-dependent replication of U_L_20-null HSV-1 (F). Electron microscopy images of this virus in non-permissive Vero cells revealed a profound entrapment of viral particles in the perinuclear space. U_L_20 is required for intracellular transport and cell surface expression of gK in transient expression experiments, indicating a role in virus-specified glycoprotein trafficking. During infection, U_L_20 protein is required for gK transport to the surface, which is necessary for virus-induced cell fusion that is caused by syncytial mutations in either gB or gK (i.e., mutations in the gB and gK genes that allow for formation of syncytia) [685]. 

Additionally, gK is a virion component that is important for virus entry into cells [686]. The role of gK during entry might stem from its interaction with the viral glycoproteins that mediate virus entry. gK forms a functional protein complex with U_L_20, which is required for gK and U_L_20-associated functions in the life cycle of HSV-1 [686,687]. Coimmunoprecipitation experiments showed that U_L_20 forms a complex with gB and gH in infected cells but not with gD [688]. Additionally, gK has a functional amino-terminal domain [689] that can interact with the extracellular portions of gB and gH [688]. These results suggest that the gK/U_L_20 complex may modulate the fusogenic properties of gB and gH via direct physical interactions. Treatment of virions with a protease that cleaves the gB-binding domain of gK results in reduced infectivity of the treated virions [690], which further supports the presence of gK on the surface of the virion and its role in mediating virus entry. Other data further support that gK is required for proper localization of gD and gH/gL on HSV-1 assembly compartments [691]. Additionally, gK is required for gB binding to Akt during entry into neuroblastoma cells, release of calcium, and fusion of the viral envelope with host membranes. In the absence of the N-terminal functional domain of gK, entry into cells occurs through endocytosis [692]. Virus entry is therefore modulated by gK at multiple levels.

An interesting sequence of clinically relevant papers show the importance of gK in ocular infection with HSV-1 (Figure 4B). While deletion of the N-terminal domain of gK does not affect growth in Vero cells, it reduced cell-to-cell spread. Ocular infection of mice with a mutant HSV-1 that lacks the N-terminus of gK produced no significant ocular disease symptoms, versus infection with a wild-type strain. Additionally, the viral genome could not be amplified from ganglionic neurons that were infected with the mutant versus the wild-type HSV-1 [693]. Therefore, the N-terminus of gK is essential for neuroinvasiveness and herpes keratitis in the mouse ocular model. Work expanding on this paper showed that a virus lacking the N-terminal domain of gK can attach to cell surfaces of Vero cells and ganglionic axons as efficiently as wild-type HSV-1; however, the mutant virus cannot enter into the cytoplasm of ganglionic neurons [692]. These data are in agreement with data showing decreased corneal scarring in ocularly infected mice with a gK mutant virus [694].

### 6.7. U_L_21

U_L_21 is an accessory gene that encodes a 535-aa protein of the tegument. It was reported by Baines et al. to be dispensable for viral replication in cell culture. U_L_21 promotes the growth of long cellular protrusions when over-expressed in non-neuronal cells and is associated with microtubules [695]. Additionally, U_L_21 forms a complex with U_L_11, U_L_16, U_L_21, and gE in transfected cells, and is necessary for the U_L_11–U_L_16 interaction [676]. 

U_L_21 is non-essential, but viral growth kinetics with a U_L_21-null virus showed that the overall viral yield is lower [696]. Most U_L_21-interacting proteins were found to be cytoskeletal proteins expressed in the central nervous system, such as the glial fibrillary acidic protein (GFAP). The distribution of GFAP is also altered in U_L_21-null virus-infected glial cells, when compared to WT-virus-infected cells. These results suggest that U_L_21 is involved in capsid transport through interacting with cytoskeletal proteins. The altered distribution of GFAP has only been reported in glial cells, so it is not clear if U_L_21 can affect trafficking in neurons and no follow-up studies are available [696]. 

Infection with a U_L_21-null HSV-1 resulted in a delay in the onset of immediate early gene expression. Additionally, a reduced number of capsids were found in the cytoplasm after U_L_21-null virus infection although DNA-containing capsids were formed in the nucleus [697]. These data suggest that U_L_21 has an early function that facilitates viral gene expression, as well as a late function that promotes the exit of capsids from the nucleus to the cytoplasm. The early function is supported by the crystal structure of the C-terminal domain of U_L_21 [698]. Based on these studies, it is shown that U_L_21 can bind *E. coli* RNA, which suggests a role for U_L_21 in transcription or translation, but further work is needed. Regarding the late function of U_L_21, multiple empty capsids have been observed in the cytoplasm of U_L_21-null-infected cells [699]. Therefore, it was suggested that U_L_21 either retains capsids in the nucleus until they receive DNA, and disruption of U_L_21 allows empty capsids to be transported to the cytoplasm, or that U_L_21 protects DNA-filled capsids and its absence results in empty capsids in the cytoplasm [699]. 

### 6.8. U_L_24

U_L_24 is a late viral gene that is expressed as a predominantly nucleus-associated 30 kDa protein [700] but localizes to the cytoplasm as well [701]. It is encoded by mRNAs with two different 5’ ends. The majority of U_L_24 is encoded by the mRNA that contains the first initiation codon of the ORF [702]. It is unclear why U_L_24 is transcribed from different sets composing six transcripts [702]. The third initiation codon in the U_L_24 ORF leads to the expression of a protein termed U_L_24.5 with a size of 18 kDa [703]. A U_L_24.5-null HSV-1 exhibits viral growth similar to a WT virus but does not trigger dispersal of nucleolar proteins as WT [703].

A U_L_24-null virus yields mildly lower titers in cells and slightly smaller plaque sizes. Corneal infection in mice with a U_L_24-null virus results in 1 log lower viral load versus WT, but there is a 4 logs lower viral growth in the trigeminal ganglia. These data suggest that U_L_24 is important for the dissemination of HSV-1 from the cornea to the trigeminal ganglia in mice (Figure 4A) [700,704]. U_L_24 may be important for virulence in murine and guinea pig models of intravaginal infection with HSV-2; however, the similarities between HSV-1 and HSV-2 U_L_24 are difficult to assess [705].

U_L_24 is one of four genes that when mutated can confer a syncytial (syn) phenotype [701]. It is not known how mutations in the U_L_24 gene confer syncytia. Mechanistically, lack of U_L_24 during late infection results in mislocalization of gB and gD with respect to actin [701], which are proteins involved in fusion. U_L_24 mutations that confer syncytia may work through the effect of U_L_24 on the localization of these fusogenic viral glycoproteins.

U_L_24 was found through bioinformatics to contain PD-(D/E)XK endonuclease signature sequences [706]. These sequences are required for the dispersal of nucleolin that occurs during infection, since their deletion or mutagenesis prevents nucleolin dispersal that occurs normally during infection. This suggests that U_L_24 is involved in nucleolin dispersal through its endonuclease motif [707,708]. Mutating the endonuclease motif also causes one log lower viral growth in the eye and the trigeminal ganglia of an ocular mouse model, indicating that the effect of U_L_24 endonuclease function is involved in dissemination of HSV-1 from the eye to the ganglia in vivo [709]. Another nucleolar component that is dispersed due to the endonuclease function of U_L_24 is the B23 nucleolar protein [710], which is a multifunctional protein that participates in ribosome biogenesis, mRNA processing, chromatin remodeling, and maintains genome stability [711]. U_L_24 also mediates nuclear egress of HSV-1 nucleocapsids and this effect most likely occurs through the abovementioned effect of U_L_24 on dispersion of nucleolar proteins [712]. 

U_L_24 may also play a role in immune evasion, through a function unrelated to its endonuclease motif [713]. Exogenous U_L_24 can bind with the p65 and p50 components of NF-κB and prevent their translocation to the nucleus. Therefore, it impairs the production of IFN-β and pro-inflammatory chemokines and may mediate immune evasion during HSV-1 infection [713].

### 6.9. U_L_31 and U_L_34 

pU_L_34 is a type 2 integral membrane protein with a 247-aa nucleoplasmic domain that binds pU_L_31 and holds it in close approximation to the inner nuclear membrane (INM) [714,715]. U_L_34 is anchored to the INM by a C-terminal transmembrane helix, with several residues extending into the perinuclear space [714]. U_L_34 retention at the INM requires the presence of U_L_31 [716], and both U_L_34 and U_L_31 localization is dependent on their co-expression and interaction [562]. U_L_31 and U_L_34 form a complex, which has been termed the nuclear egress complex (NEC), and it is required for efficient exit of nascent HSV-1 capsids from the nucleus (Figure 3A). 

Deletion of U_L_31 results in 3–4 logs lower viral yields compared to WT HSV-1, slightly decreased levels of total viral DNA, and a 3–5-fold reduction in the ratio of monomeric to concatemeric DNA, suggesting minor roles in both DNA replication and processing or packaging of viral DNA [717].

Deletion of U_L_34 results in 2–5 logs lower yields of HSV-1 in cell culture. While a U_L_34-null virus can assemble DNA-containing capsids, they accumulate in the nucleus and are unable to bud through the inner nuclear membrane [718].

The role of U_L_31 and U_L_34 initiates with the disruption of nuclear lamina during HSV-1 replication. Formation of HSV-1 replication compartments (RCs) and annexation of space in the nucleus results in cellular chromatin marginalization and compression [719]. The phase of chromatin marginalization occurs during the initial phase of RC formation, and this does not require U_L_31 and U_L_34. However, later during infection, RCs penetrate the host chromatin and the nuclear lamina, and reach a region of the nucleus close to the INM. In co-transfection experiments of U_L_31 and U_L_34, marginalization of host chromatin was not observed; however, during infection, both U_L_31 and U_L_34 are required for alteration of the distribution of lamina components [720], which suggests that other viral proteins cooperate with NEC for lamina disruption. Nonetheless, U_L_34 can interact directly with lamin A/C in vitro [721]. This disruption of lamina is a regulated process during infection since the viral protein U_S_3 is involved [557,721,722]. The kinase activity of U_S_3 was not necessary for the redistribution and disruption of lamin A/C or lamin B [559], indicating that U_S_3 spatial interaction with NEC may be the regulatory mechanism. However, U_S_3 phosphorylates lamin A/C during HSV-1 infection [558].

The N-terminus of pU_L_31 also harbors multiple phosphorylation sites of the viral U_S_3 kinase. Preventing the phosphorylation of pU_L_31 mimics the growth defect of a U_S_3-null virus, with 1-2 logs lower viral yields. The importance of the N-terminus and its phosphorylation is highlighted by the following observations: First, pU_L_31 that lacks the N-terminus is retained in the cytoplasm if co-expressed with U_L_34, suggesting that they prematurely interact before they enter the nucleus. This is probably why U_L_31 and U_L_34 utilize different transport routes to the nucleus, averting their premature interaction. Second, the phosphorylation of the N-terminus of pU_L_31 is necessary for the proper localization of the pU_L_31/pU_L_34 complex in the nuclear rim and the optimal egress of virions from the perinuclear space [560]. U_L_31 and U_L_34 distribution is even across the nuclear rim, but this requires U_S_3 expression [562]. In the absence of U_S_3, U_L_31 and U_L_34 localize in small punctate areas at the nuclear rim. This supports that U_L_31 and U_L_34 form a complex that accumulates at the nuclear membrane and plays an important role in HSV-1 nucleocapsid envelopment at the inner nuclear membrane. Absence of U_S_3 causes accumulation of capsids in nuclear membrane invaginations, delayed onset of virus production, and reduced virus titers [556]. U_L_31 and U_L_34 associate with perinuclear virions but not with extracellular virions, supporting the de-envelopment/re-envelopment model of viral egress [556]. 

NEC by itself is sufficient to drive the vesiculation of the nuclear envelope in transfected cells in the absence of any other viral proteins [723,724,725]. Using purified HSV-1 NEC components and synthetic liposomes, it was shown that NEC has an intrinsic ability to vesiculate membranes in vitro [726]. NEC formed a coat-like hexagonal lattice on the inner surface of the budded vesicles, which suggested that it vesiculated membranes without the help of other proteins by creating a hexagonal scaffold inside the bud [727]. Further structural characterization showed that HSV-1 U_L_31 and U_L_34 form the NEC heterodimer through extensive interactions that involve residues distributed throughout U_L_31 and U_L_34. The heterodimers are further organized to form oligomeric structures. Mutagenesis of their oligomeric interfaces reduced NEC-mediated budding in vitro, supporting that NEC oligomerization drives capsid budding during nuclear egress of herpesviruses [726]. However, other cellular and viral factors may still participate in NEC oligomerization and nuclear budding in cells, such as U_S_3 mentioned above. 

Besides NEC oligomerization on the INM, a host factor mediating primary envelopment of HSV-1 is the endosomal sorting complex required for transport-III (ESCRT-III). ESCRT-III promotes primary envelopment by mediating scission during HSV-1 budding through the INM [728]. U_L_34 interacts with ALIX but not other ESCRT-III proteins, suggesting that ALIX acts as an adaptor for the recruitment of ESCRT-III proteins by U_L_34, resulting in scission of the budding vesicles formed by the NEC. The mechanism of fusion of the primary envelope with the outer nuclear membrane is still not clear.

U_L_31 also functions in another interesting manner in order to conserve viral resources. Three major types of HSV-1 capsids have been described, the empty capsids (A capsids), capsids that lack viral DNA (B capsids), and viral DNA-containing capsids (C capsids) [729]. Type C capsids are preferentially selected compared to A and B to undergo primary envelopment at the INM, and the mechanism of their selective involvement involves U_L_31, U_L_17, and U_L_25. U_L_17 and U_L_25 interact and form a stable complex. The different types of capsids contain different copies of this complex. C capsids contain 75 copies, while B capsids contain 25 copies [730]. Because of its enrichment in C capsids, the U_L_25/U_L_17 complex is termed C capsid-specific complex (CCSC). While it is possible that the CCSC binds more efficiently to C capsids, an interaction has been identified between U_L_31 and CCSC in infected cells [731]. This supports a model of egress in which the CCSC is added to capsids after DNA is inserted and engages U_L_31 either in the nucleus, or within the NEC at the INM. The end result is an elegant way to conserve cellular resources by selecting only capsids that have the potential to produce infectious virions for primary envelopment [731]. 

### 6.10. U_L_35 (VP26)

The HSV-1 U_L_35 gene encodes for a 12-kDa capsid protein designated VP26, which is located on the outer surface of the viral capsid, on the tips of the hexons that constitute the capsid shell [732]. The HSV-1 capsid has an icosahedral structure, and the major capsid protein is VP5. VP5 forms both the pentons and the hexons of the capsid, which are composed of five or six VP5 monomers [733]. Pentons are located at the icosahedral vertices, while hexons form the faces and the edges of the capsid structure. VP26 is attached to VP5 molecules that make up the hexons. The C-terminus of VP26 interacts with the upper domain (UD) of VP5 [734], and their interaction has been well characterized [734,735]. VP5 and VP26 interaction is required for localization of VP26 to the sites of capsid assembly in the nucleus [736]. However, in vitro reconstitution of HSV-1 capsids showed that VP26 is not required for proper capsid assembly [732,737]. 

These data agree with reports that show that VP26 is non-essential for viral growth in vitro [738] but influences the production of infectious virus in vivo. In a mouse ocular infection, a U_L_35-null virus yields 2-fold less virus in the eye but 30–100-fold less virus in the trigeminal ganglia (Figure 4A). VP26 does not seem to affect the transport of the virus from the eye to the ganglia but is important for the replication of the virus in the ganglia [738]. Similar results have shown in vitro that a U_L_35-null virus exhibits reduced viral yields when cultured in neuroblastoma cell lines. A potential reason is mislocalization of the main capsid protein VP5, which exhibits a punctate distribution during U_L_35-null virus infection as opposed to a diffuse distribution during a WT infection [739]. 

Another way that VP26 can affect replication is by mediating incorporation of U_L_25 into nucleocapsids and by extension affecting DNA packaging. This is because U_L_25 is part of the 3-component viral terminase complex, which transports the HSV-1 genome into the viral capsid [740]. This is supported by yeast-two-hybrid data that show interaction of VP26 and U_L_25 [735].

VP26 may also affect capsid delivery to the nucleus following entry of the virus into the cells by interacting with the dynein light-chain subunits DYLNT1 and DYLNT3. Cytoplasmic dynein is a molecular motor that is associated with microtubules, and each dynein complex contains two copies of either DYLNT1 or DYLNT3. VP26, DYLNT1 and DYLNT3 colocalize with microtubules, and VP26 is required for migration of HSV-1 capsids towards the nucleus [741]. These data suggest that VP26 is important for the retrograde transport of HSV-1 capsids from the plasma membrane towards the nuclear membrane after viral entry into cells. Further work described the N-terminus of VP26 as a binding region for the dynein light-chain subunits DYNLT1 and DYNLT3 [742]. 

### 6.11. U_L_43 

U_L_43 has a size of about 32 kDa and based on its sequence, it may contain seven transmembrane domains composed almost entirely of alpha helixes [743]. U_L_43 is not present in mature extracellular virions [744]. Other functions remain unknown.

### 6.12. U_L_44 (gC)

gC is a 511-aa type I integral membrane glycoprotein that mediates HSV-1 attachment to host cell surface glycosaminoglycans. Absence of gC results in reduced binding of virus to cells, although the virus that binds can enter cells and initiate infection [745]. gC can bind to both heparin and heparan sulfate. The binding that occurs in the absence of gC is dependent on cell surface heparan sulfate [745,746]. It was shown that two areas of gC participate in heparan sulfate binding (R143, R145, R147, T150, G247). Synthetic peptides that corresponded to these two areas prevented virus binding and entry in cells, and they also agglutinated red blood cells [747]. These data suggest that these gC areas mediate the binding of virus on cell surface heparan sulfate.

Additionally, gC can regulate cell entry and infection by a low-pH pathway [748]. The presence of gC confers a higher pH threshold for acid-induced changes in gB, affecting fusion. Using a gC-null virus, it was found that there was a delay in entry relative to WT HSV-1 [745]. A research group tested infection with HSV-1 and gC-null HSV-1 on different cell lines. They observed that a gC-null virus displayed different infectivity, depending on whether these cell lines support low-pH or pH-neutral entry. Treatment with ammonium chloride did not affect gC-null HSV-1 entry into cells that support the pH-neutral pathway, suggesting that gC is dispensable for that pathway. When they assessed for infectious virus after infection in a low-pH environment, gC-null HSV-1 was lagging in intracellular transport or release from intracellular vesicles formed after endocytic entry. After treating HSV-1 ΔgC versus WT HSV-1 virions with different pH solutions, it was shown that the presence of gC increases the pH at which fusogenic conformational change of gB occurs [748]. 

### 6.13. U_L_45

U_L_45 is a late gene [749] that encodes an 18-kDa protein that is present in virions and is enriched in the envelope–tegument interface, thus associated with the viral envelope [749]. While it is non-essential for viral growth in vitro [750], it is required for efficient growth in the central nervous system (CNS) of mice when inoculating with a low viral dose [751].

U_L_45 is required for syncytia formation during infection with a gB mutant HSV-1 that causes syncytia (gBsyn) [752]. A UL45 C-terminus truncation prevents the formation of syncytia with gBsyn [753], suggesting that U_L_45 affects entry that requires gB function. However, U_L_45 plays a dispensable role in virus entry to cells either through pH-dependent endocytosis or pH-independent mechanisms [754]. 

### 6.14. U_L_55 and U_L_56

The U_L_56 gene product is a C-terminal-anchored type II membrane protein conserved among HSV-1, HSV-2, and herpes B virus. Even though U_L_56 is dispensable for viral growth in cultured cells, it plays an important role in the pathogenicity of HSV-1. 

HSV-1 mutants lacking U_L_56 are substantially less pathogenic in mice but have similar growth in cell culture [755]. The role of U_L_56 in the severity of HSV-1 ocular disease can be seen in work based on a quantitative trait locus (QTL)-based assay, which involved infecting mice with 40 recombinant strains derived from mice infected simultaneously with two avirulent strains. Phenotypically meaningful variations could be seen in multiple genes, including U_L_56, whose features were associated with an increase in ocular virulence in mice [756]. However, U_L_55 and U_L_56 do not appear to have a role in the latent stage of the virus since mice that were infected with HSV-1 lacking U_L_55 and U_L_56 could still develop a latent infection [757]. Furthermore, HSV-1 mutants lacking the entire U­_L_56 gene have been found in human samples [758] and are considered to be less pathogenic or to lack neurovirulence [755,758]. Therefore, U_L_56 is non-essential in cell culture, and is not implicated in latency but is important for pathogenicity in vivo.

U_L_56 contains a hydrophobic domain in its carboxyl-terminal tail (aa 217–234) which is embedded in the membrane, and its deletion results in reduced pathogenicity as it generates an avirulent HSV-1 strain [759]. Characterization of U_L_56 through immunofluorescence studies showed that U_L_56 localized to the Golgi and cytoplasmic vesicles in U_L_56-transfected or HSV-2-infected cells. The C-terminal domain is important for association with cytoplasmic membranes and the N-terminal is important for its translocation to the Golgi and the cytoplasmic vesicles. Protease digestion assays combined with fractionation through sucrose gradients suggested that U_L_56 is a type II membrane protein associated with lipid rafts. These data suggest that U_L_56 may be involved in vesicular trafficking in HSV-2-infected cells. Expanding on that work, an interaction between U_L_11 and U_L_56 was identified [760], suggesting a complex that may be involved in the cytoplasmic envelopment of HSV.

Ushijima et al. in a series of papers investigated the role of U_L_56 during HSV-2 infection. They first demonstrated that U_L_56 interacts through its PY motifs with Nedd4, an E3 ubiquitin ligase. U_L_56 triggered increased NEDD4 ubiquitination and its subsequent degradation during infection [761]. Additionally, they investigated potential co-localization of U_L_56 with Nedd4 and they found that U_L_56 localizes to the TGN and early endosomes but not with CD63 in late endosomes. Co-localization of U_L_56 with Nedd4 was observed at the TGN, but a lack of co-localization with CD63 suggested that HSV-2 is not using MVBs for cytoplasmic envelopment. Nonetheless, deletion of U_L_56 restricted infectious HSV-2 release. Therefore, Ushijima suggested that U_L_56 functions in coordination with other host or viral factors in trafficking and membrane sorting. Nedd4 may be among these factors, and this function of U_L_56 may be redundant with other viral proteins that have sorting functions and may also depend on the cell type [762].

Ushijima et al. in 2010 showed how U_L_56 interacts with Itch, which is another Nedd4-family ligase, triggering its degradation through lysosomes. Interestingly, HSV-1 does not degrade Nedd4, but it does degrade Itch [763].

### 6.15. U_S_2

The U_S_2 gene of HSV is predicted to encode a 291-aa protein of 33 kDa. It is predicted to have a hydrophobic N-terminus [764]; it is non-essential in cell culture and not involved in the pathogenesis in the CNS of mice [765]. HSV-1 Us2 is not associated with any specific phenotype [766]; however, most research so far has been done on HSV-2 Us2. Initially, U_S_2 was observed as discrete granules late during infection within and at the periphery of the nucleus [767]. However, further analysis of U_S_2 by immunofluorescence microscopy of infected Vero and A431 cells detected a filamentous-like cytoplasmic pattern [768]. Additional data suggested interaction of U_S_2 with cytokeratin 18 through yeast-two-hybrid assays, and confirmed the interaction by co-immunoprecipitation, which seems to involve the N-terminus of U_S_2 [768]. Other HSV gene products can also interact with proteins of the cytoskeleton [769,770,771]. U_S_2 and cytokeratin 18 interaction suggests participation of U_S_2 in trafficking during infection, but more work is needed to characterize this function.

It was later shown that HSV-2 U_S_2 is a membrane-associated ubiquitin-interacting protein [772]. HSV-2 U_S_2 lacks specific membrane sorting signals, and can be found at the plasma membrane, in cytoplasmic vesicles, and diffusely throughout the cytoplasm. Through a discontinuous gradient, U_S_2 can be detected in detergent-resistant membranes, and cofractionates with caveolin-1 and ganglioside GM1. Treatment of infected cells with BFA (brefeldin A) did not affect the localization of U_S_2, suggesting that it is not part of the ER-Golgi secretory pathway [772]. Co-localization experiments showed that U_S_2 localizes predominantly to recycling endosomes and the plasma membrane, but the resistance to BFA treatment suggests that this localization of U_S_2 is regulated post-translationally. Through mass spectrometry, most U_S_2-interacting proteins were shown to be ubiquitinated. U_S_2 could be pulled down by mono-ubiquitin conjugated agarose but not by protein G agarose, suggesting that U_S_2 interacts specifically with ubiquitin and not with ubiquitin-conjugated proteins [772].

Lu et al. in 2017 showed that HSV-2 U_S_2 could activate NF-κB signaling. Deficiencies in U_S_2 decreased HSV-2 WT-mediated NF-κB activation and cytokine and chemokine production, while overexpression of U_S_2 produced the opposite effects. Co-immunoprecipitations suggested that U_S_2 interacts with TGF-β-activated kinase 1 (TAK1). U_S_2 induced the phosphorylation of TAK1, resulting in the activation of TAK1-mediated downstream signaling. This role of U_S_2 in NF-κB activation was confirmed in mice. Interestingly, HSV-1 U_S_2 did not activate NF-κB like HSV-2 U_S_2 [773].

### 6.16. U_S_4 (gG)

Glycoprotein G (gG) is one of the least well-characterized glycoproteins of HSV-1. Infections with gG-null HSV-1 exhibit similar growth to WT virus and little attenuation in vivo [774]. When focusing on polarized epithelial cells though, gG seems to be required for infection through the apical surface. However, a gG-null virus can still infect these cells through the basal membranes and replicate normally. In vivo infection of apical surfaces of mouse corneas with a gG-null HSV-1 results in delayed scarification, but once scarification occurs, a gG-null virus has yields similar to wild-type virus [775]. 

An interesting observation is that inoculation of mice with a baculovirus recombinant vector carrying the gG ORF results in partial protection from lethal challenge with intraperitoneally injected HSV-1 [776,777]. This protection was not observed when using vaccinia virus as a vector [778]. This might be a result of higher expression of gG in the baculovirus system, or more intriguingly, gG in insect cells of the baculovirus system may be glycosylated in a different pattern that increases gG immunogenicity. However, this protection does not apply to corneal infection with HSV-1 after vaccination with the same gG-containing baculovirus vector [779].

The most interesting role of gG regards its interplay with chemokines. Chemokines are chemotactic cytokines that coordinate the recruitment of immune cells to infection sites, thus are important for the outcome of a viral infection [780]. Mice that are depleted of chemokine ligands or receptors are highly susceptible to genital herpes infection and neuroinvasion of the CNS due to defective leukocyte mobilization to the infected mucosa [781].

HSV-1 gG localizes on the plasma membrane [782], and it can bind chemokines with high affinity [783,784]. Binding of HSV-1 gG to chemokines while being on the plasma membrane of infected cells occurs through the glycosaminoglycan (GAG)-binding domain of the chemokine, which is required for binding to gG. Interestingly, binding of gG to chemokines does not inhibit chemokine function, rather increases it both in vitro and in vivo. Experiments show that higher migration of leukocytes occurs in the presence of gG on HSV-1-infected cells, due to increased chemokine binding to its specific receptor and downstream MAPK signaling mediated by the surface gG. It is possible that gG acts as a GAG and mediates a local increase of chemokine concentration in parts of the membrane. This will increase chemokine signaling, which will be beneficial for the virus in a number of ways [783]. First, it is possible that chemokine deregulation due to binding to gG promotes viral dissemination through MAPK signaling and NF-κB activation, which enhances viral replication [785]. An alternative hypothesis is that the increased infiltration of leukocytes to sites of infection increases the number of available cells that can then be infected by HSV-1, thus helping the virus spread in vivo [783]. The increased leukocyte migration hypothesis may still occur even through chemokines binding to HSV-1 particles, since gG is also present on the viral envelope [782,786].

### 6.17. U_S_5 (gJ)

U_S_5 is a late gene that encodes the glycoprotein J (gJ) of HSV-1. gJ localizes to multiple membrane compartments and has been little characterized [787]. gJ is another glycoprotein that mediates protection from CTL killing of infected cells. CTLs kill targets in part by inducing apoptosis either though activation of the Fas pathway and downstream activation of caspases or by releasing lytic granules that contain granzyme B, which can induce apoptosis by cleaving caspases in target cells. gJ can inhibit both pathways; however, other viral genes can compensate for deletion of gJ [788]. gJ can also bind the F_0_F_1_ATPase synthase in the mitochondrial membrane that is required for induction of ROS, which suggests that gJ may inhibit F_0_F_1_ATPase function [788]. 

### 6.18. gE/gI (U_S_8/U_S_7), U_S_9

Glycoprotein E (gE) was first described as a receptor for the Fc portion of immunoglobulin G and for its role in virus spread from cell to cell [789]. Glycoprotein I (gI) is the other polypeptide of the gE/gI complex [789], and like gE, it contains a 400-aa extracellular (ET) domain and a 100-aa cytoplasmic (CT) domain. Since the majority of gE is bound to gI, these proteins are often studied in tandem. 

gI can increase the affinity of gE to IgG. gE and gI mutants exhibit a small plaque phenotype in vitro compared to WT HSV-1 [774]. The number of plaques detected is not affected, suggesting that gE and gI regulate cell-to-cell spread in vitro [790]. Expression of gE/gI in human epithelial cells resulted in the localization of gE/gI at lateral surfaces of cells and colocalization with the adherens junction marker β-catenin. At subconfluent monolayers during infection, gE/gI localize at the parts of the plasma membrane that are in contact with another cell [791]. Therefore gE/gI seems to mediate cell-to-cell spread of HSV-1 across cell junctions by interacting with cell junction components (Figure 3D).

Normally, HSV-1 particles are sorted to cell junctions, whereas few virions reach the apical surfaces of polarized epithelial cells [792]. Deleting gE results in HSV-1 virions that cannot translocate to cell junctions and they leave the cell through the apical surface [792]. Work that has characterized a panel of gE mutant viruses with small insertions in the ET domain underlined the importance of this domain of gE for cell-to-cell spread. Several of these gE ET mutant proteins were able to complex with gI and be incorporated into virions, but the formed virions behaved similarly to gE-null mutants. This suggests that gE/gI promotes HSV-1 cell-to-cell spread by binding either extracellular ligands through the gE ET or components of cell junctions (Figure 3D). 

Antibodies specific for HSV-1 antigens can simultaneously bind at the surface of infected cells to gE/gI via their Fc region and to a cell surface HSV-1 antigen by their antigen-binding fragments (Fabs) [793,794,795]. This process is known as antibody bipolar bridging (ABB), and may be a strategy to prevent the host from utilizing anti-HSV-1 antibody responses. 

HSV-1 gE mutants show decreased neurovirulence [796], and the Fc binding portion of gE was shown to be important in vivo [797]. Introducing insertions in HSV-1 either inside or outside the Fc binding portion of gE resulted in lower yields only for the Fc domain mutant, when testing the two mutants in mice. Additionally, the Fc-binding gE mutant virus was impaired in its ability to reach the ganglia (Figure 4A) [797]. HSV-1 gE/gI-null mutants also show significantly reduced spread in the corneal epithelium of infected mice, due to the reduced ability of these mutants to undergo anterograde transport from sensory ganglia back to the cornea [798].

A major factor for the decreased neurovirulence and cornea infection of gE mutants is the less efficient anterograde transport (Figure 4A). Anterograde transport in infected neurons (e.g., after reactivation of virus from latency) involves the transport of viral particles from the neuronal-cell body along axons to axonal termini, and transfer across junctions formed between neurons and epithelial cells. gE and gI mutants displayed markedly reduced anterograde spread between neurons within the retina and from the retina to retinorecipient regions of the brain [799]. HSV-1 gE/gI and U_S_9 possess overlapping or additive effects in anterograde axonal transport. Mutants lacking gE, gI, or U_S_9 displayed significantly reduced transport of capsids and glycoproteins towards axonal termini, while concomitant deletion of gE and U_S_9 produced nearly zero levels of capsids and glycoproteins even in proximal axons [800,801]. Investigation of HSV-1 infection using a mouse retina model concluded that HSV-1 U_S_9 is required for the transport of capsids, but not viral glycoproteins, from the retina into the optic nerve (Figure 4D) [802]. U_S_9 is a viral tegument protein [803] that has been found associated with the ER and the Golgi [802], and with unenveloped capsids. It is tail anchored, has no ectodomain, and contains a cytoplasmic domain with TGN localization signals, which are important for its function [804,805]. 

The fact that gE and U_S_9 HSV-1 mutant viruses accumulate in the cytoplasm and do not enter the axons suggests either a trafficking issue or defective virion envelopment. Further characterization of the assembly of those mutants showed accumulation of unenveloped capsids in the cytoplasm of the infected cells and few enveloped virions. Both cannot enter axons in neuronal cells. Additionally, most capsids produced from gE and Us9 mutant viruses remained adhered to, or nested up against, membranes in the cytoplasm [806]. Considering that gE/gI and U_S_9 accumulate in the TGN (a site of virus assembly), and that the gE/gI complex sorts virus particles to epithelial cell junctions [792], the loss of gE/gI and U_S_9 might lead to misrouting of HSV-1 capsids and virions so that they do not enter axons. Alternatively, the defective cytoplasmic envelopment might also inhibit anterograde transport. These defects may depend both on gE and U_S_9 since U_S_9 has been shown to colocalize with capsids and not glycoproteins, whereas gE/gI colocalized with glycoproteins and not capsids [800].

It was also reported that gI can induce the formation of rod-shaped structures [807]. About 40% of gI-transfected cells expressed rod-shaped structures in the cytoplasm, besides the typical gI localization patterns (nuclear rim and cytoplasmic speckles, and junctions). The rods themselves vary in width and length. By doing immunofluorescence analysis using antibodies against different domains of gI, it was shown that the aa 110–202 of gI in the rod-shaped structures are not exposed. Since gE interacts with the aa 128–145 of gI, it will not interact with the gI of the rod-shaped structures, suggesting that gE is important for the proper function of gI. Two proline residues have been implicated in the induction of the gI rods, but they are not required for viral replication. However, these residues are important for mediating syncytia formation during infection with a U_L_24 mutant virus [807]. Thus, the coordination between gI and U_L_24 may be important for viral cell-to-cell spread and pathogenesis. 

### 6.19. U_S_8.5

The U_S_8.5 gene overlaps with parts of the U_S_8 and the U_S_9 gene [808]. Its transcription is initiated within the coding sequence of U_S_8 and it is transcribed earlier than U_S_8, while the U_S_8.5 transcript is co-terminal with the transcripts of U_S_8 and U_S_9 [809]. The U_S_8.5 protein localizes in the nucleoli, but its function remains unknown. 

### 6.20. U_S_10

The U_S_10 gene encodes a polypeptide of 313 aa [764] that was identified as a capsid/tegument-associated protein localizing to nuclei as foci late during infection [810]. Deletion of U_S_9, 10, 11, or 12 has no effect on the neurovirulence and latency/reactivation potential of HSV-1, but it affects its neuroinvasiveness from peripheral sites to the CNS [810]. Fractionation studies show U_S_10 tightly associating with the nuclear matrix. Analysis of isolated intracellular capsids showed that both phosphorylated and unphosphorylated forms of U_S_10 were associated with the capsid/tegument. The nature of this association remains to be determined.

### 6.21. U_S_11

U_S_11 was identified as a late gene since it was shown that its expression required DNA replication [811]. Early work described the RNA-binding activity of U_S_11 to an in vitro RNA transcript of HSV-1 [812], which was later shown to be the U_L_34 transcript. U_S_11 binding to U_L_34 mRNA prevented its accumulation [813]. Expression of U_S_11 in baby hamster kidney cells prevented HSV-1 infection through a gD-mediated step, but this effect was not clarified [814]. Simonin et al. in 1995 [815] showed U_S_11 is phosphorylated independently of viral genome expression, by host kinases. The first work that connected U_S_11 and PKR showed that U_S_11 can bind protein kinase R in vitro and preclude the phosphorylation of eIF-2α [319]. Work by the same group showed that U_S_11 and PKR interact in the context of viral infection and this interaction is RNA dependent (Figure 2D) [816]. 

Us11 is an abundant tegument protein of the virus that is released in the cells during virus entry. It can later be found in ribonucleoprotein fibrils, clusters of interchromatin granules, and in nucleoli [817]. Importantly, U_S_11 distribution in the nucleus follows that of nucleolin. Additionally, nuclear egress of HSV-1 capsids requires nucleolin, and U_S_11 and nucleolin associate during infection. The polyproline type II helix-containing domain of U_S_11 is required for this interaction, and this domain is also responsible for U_S_11 nucleolar accumulation. Nucleolin is involved in nucleocytoplasmic shuttling and U_S_11 accumulates in the nucleolus in its absence [817]. These data suggest that nucleolin could regulate the nucleocytoplasmic shuttling of U_S_11 during infection.

Interesting work showed that U_S_11 protein can bind HTLV-1 and HIV-1 responsive elements and can transactivate envelope retroviral glycoprotein expression by binding to the Rex-responsive element (RexRE), which is located in the 3’ untranslated region (UTR) of the HTLV-1 *env* mRNA [818]. Such data raise the possibility of in vivo interactions between herpes virus and human retroviruses, possibly affecting the expression of each virus at the post-transcriptional level. Further work showed how U_S_11 binds to HSV-1 mRNAs, such as the HSV-1 U_L_34 mRNA (its natural target), which results in its accumulation and might affect its trafficking. Two different U_S_11 domains were described, a C-terminal RNA-binding domain and an N-terminal effector domain, the deletion of which created a trans-dominant negative mutant [819]. 

More work on the binding partners of U_S_11 identified binding between the RNA-binding domain of U_S_11 and a 600-bp RNA sequence that is present in the co-terminal HSV-1 mRNAs U_L_12, U_L_13, and U_L_14 [600]. U_S_11 downregulates expression of the U_L_13 protein kinase at early times during infection [600]. U_L_13 is expressed with late gene kinetics but is also a component of the tegument. In the absence of U_L_13, there is a decrease in the accumulation of a subset of late mRNAs. U_S_11 might downregulate U_L_13 during early infection to stall the accumulation of the late mRNAs. These data further support the post-transcriptional control that U_S_11 applies to viral transcripts.

The subject of multiple studies has been the interaction of U_S_11 with PKR. The PKR kinase is an RNA sensor that upon sensing viral RNAs, phosphorylates the alpha subunit of the translation initiation factor eukaryotic initiation factor 2 (eIF2α) and thereby inhibits protein synthesis. The viral proteins γ_1_34.5 and U_S_11 prevent the accumulation of phosphorylated eIF2α and consequently the translational shutoff. Particularly, the γ_1_34.5 protein directs protein phosphatase 1α to dephosphorylate eIF2α, reversing the effects of PKR activation (Figure 3D). Us11 when expressed under an immediate early promoter can rescue the growth of a γ_1_34.5-null virus. This requires a 68-amino-acid fragment of U_S_11, which contains the RNA binding domain and was found to be sufficient for preventing PKR activation, thereby allowing protein synthesis and rescuing the growth of γ_1_34.5 mutant viruses [820]. This 68-aa domain of U_S_11 can also inhibit activation of PKR in a cell-free system, supporting the RNA binding function that has been ascribed to U_S_11. Through its interaction with PKR, U_S_11 can also inhibit autophagy activation by dsRNAs in a Beclin-1-independent manner [821]. 

The protein activator of PKR (PACT) is another restriction factor for HSV-1 as HSV-1-induced interferon production in murine cells was inhibited in the absence of PACT. Binding of PACT to PKR is a dsRNA-independent mechanism of activation of PKR. PACT-mediated PKR activation occurs only under cellular stress, such as withdrawal of growth factors or treatment with a low dose of actinomycin D [822]. U_S_11 can prevent PACT-mediated PKR activation by binding to the dimerization domain (DD) of PKR. This allows binding of PACT to PKR but prevents the conformational change of PKR that is normally induced by PACT and activates PKR [823]. U_S_11 was found to bind both to PKR and to PACT, but only its binding to PKR was essential for preventing PACT from activating PKR, although binding of PACT to PKR was not prevented [824]. Further work on the binding of U_S_11 to PACT suggested that it might also prevent PACT-mediated activation of RIG-I (Figure 2D) [824].

U_S_11 also has a proviral role through inhibiting the synthesis of 2’-5’ oligoadenylate by the oligoadenylate synthase (2’-5’-OAS) (Figure 2D). 2’-5’-OAS is another dsRNA sensor that acts to block protein synthesis and decreases RNA stability in virus-infected cells. Following RNA binding, 2’-5’-OAS can synthesize 2’-5’ oligoadenylates (OA) from ATP that activate RNase L. Subsequently, RNase L cleaves mRNAs and rRNAs, inhibiting virus infection. HSV-1 can inhibit OA synthesis in IFN-stimulated primary human cells through the action of U_S_11. This inhibition requires the RNA-binding motif of U_S_11, which suggests that the mechanism involves partitioning of RNAs during infection [825].

U_S_11 can also counteract type I IFN activation by binding to Hsp90 and preventing TBK1 binding (Figure 2B). Consequently, downstream IRF3 phosphorylation and IFN induction are inhibited [826]. Further work from the same research group showed that U_S_11 also binds to the tripartite motif protein 23 (TRIM23), which is a key regulator of autophagy-mediated antiviral defense mediated by TBK1. The formation of autophagosomes mediated by TRIM23 or TBK1 is reduced by U_S_11 in infected cells, through the exclusion of TBK1 from the TRIM23 complex in infected cells, in mouse embryonic fibroblasts (Figure 2C) [826]. It should be noted, however, that the exclusion of TBK1 might depend on cell type, especially since TBK1 is implicated in human fibroblasts in the modulation of autophagy [827]. 

U_S_11 is also involved in anterograde transport of HSV-1 in dorsal root ganglia (DRG). Kinesin is a microtubule-dependent molecular motor in cells that is used for transport of unenveloped HSV-1 nucleocapsids. U_S_11 was identified as a kinesin-binding protein, and an interaction between U_S_11 and the heavy chain of kinesin (uKHC) was described [828]. The 20 to 24 RXP repeats in the carboxy half of U_S_11 that bind RNA are also where uKHC can bind. This polyproline domain may acquire a type II helix conformation with arginine residues on one side that interact with the negatively charged RNA. The other side contains hydrophobic, uncharged, or acidic chains that may provide specificity to the RNA binding and contain the uKHC binding site [829]. Another U_S_11 binding protein is the cellular PAT1 polypeptide, which binds microtubules, is involved in the intracellular trafficking of amyloid precursor protein (APP), and contains a region homologous to kinesin light chain (KLC). The U_S_11–PAT1 interaction also requires the carboxy-terminal RNA-binding domain of U_S_11 [830]. This association of U_S_11 with another molecular motor-associated protein further suggests a role for U_S_11 in trafficking of unenveloped capsids.

The effect of U_S_11 on neurovirulence has been further investigated in vitro and in vivo [831]. Intracranial infection of mice with a U_S_11-null virus is pathologically like a wild-type infection. In contrast, corneal infection with a U_S_11-null virus requires a longer time for onset of morbidity, indicating a role for U_S_11 in neuroinvasion. Replication in trigeminal ganglia and periocular tissue was mediated by U_S_11. However, U_S_11 deletion does not affect latency and the frequency of reactivation from trigeminal ganglia, even though the U_S_11-null virus reemerges with slightly slower kinetics [831]. 

A possible role of U_S_11 in viral dissemination may stem from its packing in extracellular vesicles (EVs). We have detected U_S_11 in ESCRT^+^ EVs that have been isolated from cells infected with HSV-1 [225]. EVs that contain U_S_11 may reach neighboring uninfected cells and alter their status in order to regulate viral dissemination.

## 7. Conclusions

HSV-1 encodes a large set of genes that regulate different facets of the virus life cycle, such as virus entry, viral gene transcription and expression, DNA replication, virion formation and release, host evasion, and pathogenesis. Some of the non-essential proteins of HSV-1 have been studied more extensively than others, while the functions of some genes still remain unknown. Considering that the approximately 100 gene products encoded by the virus support both viral functions and host evasion simultaneously, it is mandatory that each gene product has multiple roles throughout the virus life cycle. This results in functional redundancy that contributes to the versatility of the virus. In this review, we summarized research that has been carried out on more than 50 non-essential proteins that function in different facets of the HSV-1 life cycle. 

Non-essential genes are dispensable in vitro when ablated individually, and a lot of work to delineate their functions is based on deleting or mutating them and investigating their effects on viral processes and antiviral responses. However, many non-essential proteins work as components of functional networks in complex circuits, such as U_L_11/gE/gD, U_L_16/gE/U_L_11/U_L_21/VP22, U_L_31/U_L_34/U_S_3, or ICP0/U_L_46/U_L_47/ICP22/U_S_3/U_L_13/VP22 (described above). Therefore, it is likely that the field of HSV-1 research would benefit from investigating non-essential proteins not individually, but in tandem. An example is the work that has been done on HSV-1 mutants that trigger the formation of syncytia, in which regulatory interactions among HSV-1 proteins were described based on the effect of a non-essential protein deletion in the context of a syncytial HSV-1 mutation [672,709,832]. A potential problem that may arise by mutating non-essential genes in tandem is that the resulting virus may not be viable. 

Another consideration for further research is the relevance of the model systems being used. Various non-essential proteins should be properly investigated in a relevant physiological context, depending on their function. For example, the role of non-essential proteins in cell-to-cell spread may give different insights when investigated in polarized epithelial cells, versus human fibroblasts, or the functions in trafficking will be different in neurons with long axons versus epithelial cells. Systems like organotypic brain slice cultures [833] can be used that recapitulate more closely the variable environment of a natural infection, considering that organotypic epithelial culture systems have been successfully utilized for the study of HSV-1 ribonucleotide reductase [834]. Such considerations extend to animal models as well, since the host immune regulation may be better investigated in a model other than mice. Spontaneous reactivation of latent HSV-1 occurs in humans and rabbits but not mice [835]. The human Stimulator of Interferon Genes (STING) is regulated in a different manner than the murine STING, complicating the study of non-essential protein functions on innate immunity [836,837]. It has also been described that ICP47 binding to the murine TAP is far weaker than the human one; therefore, the prevention of MHC I antigen presentation cannot be properly evaluated in mice, and this has implications for studies of neurovirulence and survival in the CNS [341,345,346]. Transgenic animal models, or alternative models, such as pigs, dogs, or monkeys [345], may have to be chosen carefully for research questions regarding the function of such non-essential proteins. 

Another possibility for recapitulating a natural infection of the human CNS is the recently established brain organoids [838]. Recent advances in stem cell differentiation permit the use of human-induced pluripotent stem cells (hiPSCs) to generate three-dimensional (3D) neuron cultures, which are referred to as brain organoids. They exhibit neuronal heterogeneity and lamina-like structure [839]. Brain organoids can be infected with HSV-1 and exhibit inflammation, HSV-1 can establish hallmarks of latency in such cultures in the presence of antivirals like interferon (IFN), and HSV-1 can infect the outer laminar structure of these organoids moving further inside after infection [840,841]. All these elements make the use of brain organoids in HSV-1 research attractive. Investigation of latency can be complemented with the use of Lund human mesencephalic (LUHMES) cells [842]. These are human embryonic neuronal precursor cells that can be differentiated to postmitotic neurons and they can be used as a supplemental model to study latency and interactions of host and viral non-essential proteins. After infection of HSV-1, there is a loss of lytic gene transcription and an increase in the number of neurons that express latency-associated transcripts (LATs) [842]. The advantage of the latter system is that it does not require the presence of an antiviral factor like IFNβ to maintain latency.

The non-essential genes of HSV-1 offer unique properties that can be utilized for the development of recombinant HSV-1 viruses suitable for oncolytic therapy [843]. The first oncolytic virus to receive regulatory approval in the United States was an attenuated HSV-1 (named T-VEC) that has been engineered to lack ICP34.5, a major HSV-1 neurovirulence factor, ICP47, which normally prevents MHC I antigen presentation, and to express the human GM-CSF gene, which promotes dendritic cell accumulation at sites of inflammation and enhances APC function [844]. T-VEC was approved for the treatment of patients with local unresectable malignant melanoma. T-VEC is currently being investigated for further potential uses against Merkel cell carcinoma, and other malignancies [845,846]. The multiple roles of HSV-1 non-essential proteins in vivo make the study of manipulating their function worthwhile for therapeutic purposes [847]. For example, engineered HSV-1 lacking the U_L_39 gene, which is required for replication in non-dividing cells (i.e., neurons), makes it an attractive candidate for targeting gliomas [848]. For similar reasons, HSV-1 mutants lacking the function of the uracil glycosylase U_L_2 [849] or U_L_56 are less neurovirulent and good candidates for further investigation. Besides oncolytic therapy, non-essential proteins can be utilized for vaccine research. An HSV-1 strain carrying a deletion in gK cannot infect neuronal axons and establish latency. This strain has been explored as a vaccine to confer protection against lethal intravaginal HSV-1 and HSV-2 challenge in mice and rhesus macaques [850,851]. ICP0-null or vhs-null viruses combined perhaps with other mutations could be explored for vaccine strategies due to their attenuated phenotype as well.

Significant research has been carried out on the roles of non-essential proteins of HSV-1 for many decades. While many aspects of the virus have been unveiled regarding the networks of interactions and the role of individual proteins in the virus life cycle, a lot remains to be clarified regarding the role of non-essential proteins in vivo. Further investigation of the non-essential HSV-1 proteins will allow us to better understand mechanisms of HSV-1 pathogenesis and disease, and will also enable effective harnessing of HSV-1 properties for cancer, gene therapy, and vaccine strategies.

## Figures and Tables

**Table 1:** Non-essential genes of HSV-1, corresponding proteins, their location on the HSV-1 virion, and their function. Pink: tegument proteins, blue: accessory proteins, yellow: envelope proteins, green: capsid proteins.

Gene	Protein	Location on Virion	Function
*RL1* or *γ134.5*	ICP34.5	tegument	Prevents host translational shutoff and autophagy
*RL2 or α0*	ICP0	tegument	Promiscuous transactivator of genes, disrupts repressor complexes, E3 ubiquitin ligase, inhibits innate immunity, modulates endocytosis, etc.
*UL2*	uracil-DNA glycosylase	accessory	nucleic acid metabolism
*UL3*		accessory	
*UL4*		accessory	
*UL7*		tegument	Virion assembly and egress
*UL10*	gM	envelope	Host and viral protein trafficking
*UL11*		tegument	Cytoplasmic envelopment
*UL12*		accessory	Nucleic acid metabolism
*UL12.5*		accessory	Involved in depleting mtDNA
*UL13*	Ser/thr protein kinase	tegument	Blocking innate immune responses, supporting viral protein synthesis
*UL16*		tegument	Cytoplasmic envelopment
*UL20*		envelope	Glycoprotein trafficking
*UL21*		tegument	Promotes capsid egress to the cytoplasm
*UL23*	thymidine kinase (TK)	tegument	Broad spectrum nucleoside kinase
*UL24*		accessory	Glycoprotein trafficking, nucleolus dispersal
*UL31*		accessory	Component of the nuclear egress complex (NEC), promotes primary nuclear envelopment
*UL34*		accessory	Component of the nuclear egress complex (NEC), promotes primary nuclear envelopment
*UL35*	VP26	capsid	Affects DNA packaging, mediates capsid assembly, trafficking post viral entry
*UL39*	RR1 (ribonucleotide reductase)	accessory	Part of the ribonucleotide reductase (RR) complex, converts ribonucleotide diphosphates to corresponding deoxyribonucleotides, allowing for virus replication particularly in non-dividing cells
*UL40*	RR2 (ribonucleotide reductase)	accessory	Part of the ribonucleotide reductase (RR) complex, converts ribonucleotide diphosphates to corresponding deoxyribonucleotides, allowing for virus replication particularly in non-dividing cells
*UL41*	VHS	tegument	Viral RNase, degrades host transcripts and blocks antiviral responses
*L43*		tegument	
*UL44*	gC	envelope	Mediates viral binding to heparan sulfate, regulates entry by a low-pH pathway
*UL45*		envelope	Required for syncytia formation during HSV-1 gB syn infection
*UL46*	VP11/12	tegument	Regulation of transcription, activates pathways for cell survival, blocks pathways for innate immunity activation
*UL47*	VP13/14	tegument	Regulation of transcription, modulating post-transcriptional processing of mRNAs
*UL49*	VP22	tegument	Facilitates viral gene expression, protein expression, and DNA replication; inhibits inflammasome
*UL49.5*	gN	envelope	Binding partner of gM
*UL50*		tegument	Nucleic acid metabolism
*UL51*		tegument	Participates in cytoplasmic envelopment; facilitates virus spread from cell-to-cell; recruits UL7 to tegument
*UL53*	gK	envelope	Participates in virion egress from host cell; regulates virus entry and fusogenic activity of the virion; complexes with UL20
*UL55*		tegument	Participates in cytoplasmic envelopment
*UL56*		tegument	Participates in cytoplasmic envelopment
*US1*	ICP22	accessory	Regulates viral late gene expression; facilitates formation of complexes important for protein folding; participates in primary envelopment; blocks immune responses
*US1.5*		accessory	Participates in viral gene transcription
*US2*		tegument	Protein trafficking
*US3*	Ser/thr protein kinase	tegument	Blocks apoptosis, enhances viral gene expression, facilitates capsid nuclear egress, phosphorylates numerous substrates
*US3.5*	Ser/thr protein kinase	tegument	Phosphorylates substrates but cannot block apoptosis and does not facilitate nuclear egress
*US4*	gG	envelope	Regulation of chemokines
*US5*	gJ	envelope	Inhibits apoptosis and cell stress pathways
*US7*	gI	envelope	Enhances virus spread from cell-to-cell; facilitates anterograde transport of virions after reactivation from latency; important for neurovirulence
*US8*	gE	envelope	Enhances virus spread from cell-to-cell; facilitates anterograde transport of virions after reactivation from latency; important for neurovirulence
*US8.5*		accessory	Localizes in the nucleoli
*US9*		tegument	Enhances virus spread from cell-to-cell; facilitates anterograde transport of virions after reactivation from latency; important for neurovirulence
*US10*		tegument	Important for neurovirulence
*US11*		tegument	Block PKR activation and shutoff of host translation; block IFN induction; regulation of virus genes expression; trafficking of unenveloped capsids
*US12*	ICP47	accessory	Prevents MHC I antigen presentation, supports neurovirulence
